# Development of Degradable Silylated Castor Oil-Based Microparticles for the Sustained Release of a Lipophilic Anti-Cancer Drug

**DOI:** 10.3390/pharmaceutics18091145

**Published:** 2026-09-11

**Authors:** Lucas M. Favre, Ezechiel Romone, Pierre Cuq, Nicolas Masurier, Anne Aubert-Pouëssel

**Affiliations:** 1 Institut des Biomolécules Max Mousseron (IBMM), Univ Montpellier, CNRS, ENSCM, Montpellier, Francepierre.cuq@umontpellier.fr (P.C.); nicolas.masurier@umontpellier.fr (N.M.); 2 Institut Charles Gerhardt Montpellier (ICGM), Univ Montpellier, CNRS, ENSCM, Montpellier, France

**Keywords:** hybrid microparticles, castor oil, sol–gel cross-linking, poorly water-soluble molecules, anti-cancer drug formulation, sustained release, enzymatic degradability

## Abstract

**Background/Objectives**: Hybrid microparticles (HMP) based on silylated vegetable oils have recently emerged as promising carriers for the sustained delivery of poorly water-soluble drugs, offering a controlled release without a burst effect and excellent biocompatibility. However, their limited degradability and the acidic conditions required for the sol–gel encapsulation process restrict their biomedical applicability, particularly for acid-sensitive active compounds. The aim of the present study was to develop new degradable vegetable oil-based HMP with improved drug-solubilization capacity while preserving compatibility with acid-sensitive molecules through an adapted mild sol–gel process, buffered with sodium citrate. **Methods**: Two new modified oils were synthesized from castor oil derivatives, including a monoglyceride (glyceryl monoricinoleate) and a polylactic acid (PLA) conjugate, to enhance both solubilization capacity and degradability. The acid-sensitive, lipophilic antitumor compound JMV5038 was used as a model drug. Modified oils were evaluated for drug-solubilization capacity, and the resulting HMPs were characterized for particle size, encapsulation efficiency, in vitro release kinetics, cytocompatibility, antiproliferative activity against melanoma cells, and in vitro enzymatic degradability. The sol–gel protocol was optimized to minimize drug degradation during encapsulation. **Results**: Microparticles (20–111 µm) exhibited a hybrid composition with efficient cross-linking. Encapsulation efficiencies of 85–100% were achieved, aided by up to 10-fold higher drug solubility in the new oils compared to original ICOe. Release kinetics were sustained and linear (11–53 days), fastest for IGRe- and slowest for ICOLAe-based HMP. Enzymatic degradation reached up to 46% after 30 days in the presence of fungal lipases, twofold higher than previously published ICOe microparticles, with ICOLAe degrading fastest due to PLA incorporation. Encapsulated JMV5038 retained its cytotoxic efficacy against A375 melanoma cells. **Conclusions**: These results highlight the promising use of such microparticles as versatile carriers for the prolonged release of lipophilic and acid-sensitive drugs.

## 1. Introduction

In the early stages of drug development, particularly during hit-to-lead screening and structure–activity relationship studies, efforts are primarily made on improving the biological activity of new chemical entities, often with limited consideration for their physicochemical properties [1]. As a result, many lead compounds exhibit high lipophilicity and low aqueous solubility, which significantly hinders their formulation for in vivo studies and therapeutic use [2,3].

Drug delivery systems, notably polymeric and lipid-based microparticles, have emerged as effective strategies for the encapsulation and sustained release of poorly water-soluble drugs [3,4]. One of their major advantages lies in their biocompatibility, their ability to form microparticles, or their capacity to act as drug reservoirs, enabling sustained drug release over extended periods. Biodegradability is an essential property for such carriers, as it ensures their complete breakdown in the body following drug release via hydrolysis or enzymatic reactions [5]. This feature has been extensively studied in polymer-based systems, which often contain hydrolyzable chemical groups such as esters, ortho esters [6,7], anhydrides [8], phosphazenes [9] or lactic-co-glycolic acid (PLGA) [10]. Lipid-based polymers, particularly those derived from vegetable oil, are gaining attention as promising carriers for the sustained delivery of poorly water-soluble drugs [11,12,13,14,15,16,17,18,19]. These materials combine lipophilic character with structural versatility, as well as being a sustainable alternative to fossil fuel-derived polymers. They can be formulated in an aqueous medium without the use of organic solvents; their physicochemical properties ensure the encapsulation of highly lipophilic drugs and complete, prolonged release. They can potentially be degraded by lipases.

Subcutaneous drug delivery systems have gained success in the last few years, as they allow for prolonged exposure to the drug, reduction in doses and frequency of administration, thereby offering greater comfort to patients, associated with fewer side effects [20]. This type of drug delivery system can be used for cancer treatments, especially skin cancer. Among them, melanoma is the most aggressive type, with only a 35% survival rate at advanced stages [21]. It originates from the malignant proliferation of melanocytes, primarily in the skin, then spreading and forming metastases in distant organs such as the lungs, liver, or brain [22]. At that metastatic stage, therapeutic solutions are limited to systemic therapy using chemotherapeutic agents (dacarbazine, carboplatin and paclitaxel), often confronted with cancer resistance [23,24,25], or immunotherapy. For patients harboring a BRAF V600 mutation, present in approximately 50% of melanoma cases, targeted therapy combining BRAF inhibitors (vemurafenib, dabrafenib) with MEK inhibitors (trametinib, cobimetinib) has significantly improved progression-free and overall survival compared to chemotherapy alone [26]. However, the clinical benefit of these targeted therapies is frequently limited by the emergence of acquired resistance, arising through reactivation of the MAPK pathway or activation of bypass signaling routes, which restricts long-term disease control [27]. When diagnosed at an early stage, surgical resection of the tumor mass on the skin surface offers great success; however, large surgical margins or supplementary radiation therapy or immunotherapy is needed to eliminate all remaining cancer cells [25].

In this context, we were interested in developing new subcutaneous drug delivery systems able to offer a sustained release of an anti-melanoma agent currently under development (JMV5038, an imidazopyridine-fused [1,3]-diazepinone molecule) after tumor excision, in order to improve overall therapeutic care. Our group recently developed castor oil/siloxane-based hybrid microparticles (HMP) compatible with subcutaneous injection, capable of efficiently encapsulating hydrophobic drugs such as estradiol [11] or ibuprofen [12]. Castor oil is a readily available non-edible vegetable oil and naturally contains hydroxyl groups along its backbone, making it suitable for functionalization followed by sol–gel cross-linking. The release kinetics of the encapsulated compounds were linear and complete with no burst effects, in contrast to conventional polymer-based systems. Moreover, the release profile could be finely tuned by adjusting the silylation ratio, highlighting the versatility of these carriers for controlled drug delivery applications. In addition, in vitro studies showed the absence of cytotoxicity of these vegetable oil-based microparticles, while in vivo subcutaneous administration proved their biocompatibility.

However, these first-generation HMPs were not biodegradable under physiological conditions, which could limit their applicability in biomedical applications, where complete degradation of the delivery system is required. In addition, the preparation of these HMPs from modified vegetable oils through sol–gel chemistry typically relies on acid-catalyzed hydrolysis and condensation of siloxane precursors. These mildly acidic conditions (typically using acetic acid at pH < 4) are essential to initiate the formation of the siloxane network and to achieve complete cross-linking within an acceptable time frame (<1 week). However, such conditions may be detrimental to acid-sensitive drugs. Exposure to low pH during the sol–gel process may lead to hydrolysis, isomerization, or loss of activity in sensitive compounds, particularly those containing fragile ester groups or exhibiting pH-dependent structural stability. Adapting the sol–gel protocol to milder conditions is therefore necessary to accommodate such drugs within vegetable oil-based systems and to extend their applicability to a broader range of active molecules. Furthermore, while castor oil is the most polar of all vegetable oils and offers valuable solubilizing power, its solubilization capacity remains limited for certain classes of drugs, making the exploration of its chemically modified derivatives a worthwhile strategy to further enhance drug loading.

To develop new vegetable oil-based microparticles with improved degradability while ensuring compatibility of the encapsulation process with acid-sensitive drugs, a mild and adapted sol–gel approach was implemented using JMV5038, a representative lipophilic and acid-sensitive compound. JMV5038 belongs to a series of imidazopyridine-fused [1,3]-diazepinone molecules that have demonstrated cytotoxic activity against multiple cancer cell lines, including several melanoma lines [28,29,30]. Physicochemical characterization of this series revealed high lipophilicity, with an experimental Log *p* value of 3.7 ± 0.3 for JMV5038, together with limited stability under acidic conditions [13].

Previous work from our group successfully formulated JMV5038 into vegetable oil-based hybrid microparticles, specifically using a silylated castor oil (ICOe). These microparticles achieved a drug loading of 2% (*w*/*w*), while preserving the biological activity of the cytotoxic agent and enabling its sustained release over 30 days [13]. Building on these promising results, the present study aims to further optimize this delivery system by enhancing the solubilization capacity of the modified oils, the preservation of the encapsulated drug, and the degradability of the resulting hybrid microparticles. A castor oil-derived monoglyceride (different from the triglyceride structure of castor oil), as well as a conjugate between castor oil and the biodegradable polylactic acid (PLA) [31] (the PLA chain being formed by the ring-opening reaction of the D,L-lactide on the free hydroxyls of CO to introduce biodegradable bridges (esters)) were synthesized and subsequently silylated to evaluate their ability to generate HMP. We hypothesized that these two new oils would ensure better drug solubilization as they have amphiphilic structures, like surfactants. We put forward the hypothesis that their degradation would be facilitated by two different mechanisms: the low degree of cross-linking, thanks to the use of the monoglyceride, would allow water and enzymes to infiltrate the HMP matrix, leading to hydrolysis of the triglyceride ester bonds, while PLA is characterized by its ability to degrade through hydrolysis of ester bonds, which would destabilize the cross-linked network. Finally, the use of a different buffer salt to catalyze the sol–gel reaction can ensure cross-linking whilst minimizing the degradation of acid-sensitive molecules. The solubility of JMV5038 in these modified oils was then measured, and drug-loaded microparticles were developed and characterized in terms of particle size, encapsulation efficiency, in vitro release kinetics, cytocompatibility, in vitro anticancer activity against a melanoma cell line, and in vitro enzymatic degradability in the presence of fungal lipases, as a proof-of-concept.

## 2. Materials and Methods

### 2.1. Materials and Reagents

Pharmaceutical-grade castor oil (MW = 934 g/mol) was purchased from Cooper (Melun, France). Glyceryl monoricinoleate was supplied by IOI Oleochemical (Hamburg, Germany). 3-(triethoxysilyl)propyl isocyanate (IPTES; MW = 247.3 g·mol^−1^), κ-carrageenan, D,L-lactide (MW = 144.1 g·mol^−1^), acetic acid (pKa 4.76), sodium acetate, citric acid (pKa 3.13, 4.76 and 6.40), sodium citrate dihydrate, ethyl acetate, tin 2-ethylhexanoate (SnOct; MW = 405.1 g·mol^−1^), Porcine Pancreas lipase Type II (PPL), *Candida rugosa* Lipase Type VII (CRL) and solvents were supplied by Sigma-Aldrich (Saint-Quentin-Fallavier, France). 3-amino-7-dimethylamino-2-methylphenazine hydrochloride (neutral red), 3-(4,5-Dimethylthiazol-2-yl)-5-(3-carboxymethoxyphenyl)-2-(4-sulfophenyl)-2H-tetrazolium (MTS) and Dulbecco’s Modified Eagle Medium (DMEM) were used for cell studies and were also supplied by Sigma-Aldrich (Saint-Quentin-Fallavier, France).

A375 human melanoma cancer cell line for the cytotoxic activity assays and NIH 3T3 fibroblast cell line for the cytocompatibility assays were both obtained from ATCC.

### 2.2. Synthesis of JMV5038

JMV5038 was synthesized according to our previously reported procedure and its spectral data were consistent with those reported in the literature (purity > 99%, MW = 369 g·mol^−1^) [32].

### 2.3. Preformulation Study of JMV5038

The partition coefficient (n-octanol/water) of JMV5038 was experimentally determined following the OECD (Organization for Economic Co-operation and Development) guidelines for the testing of chemicals [33], and the concentration C of the compound in each phase was determined by HPLC thanks to a protocol described below. Then, the partition coefficient (P) was calculated following Equation (1).

(1) $$P=\frac{C_{octanol}}{C_{water}}$$

The stability of JMV5038 in different buffers was studied as follows: a stock solution of JMV5038 (1 mg·mL^−1^) in methanol was prepared and diluted to a final concentration of 25 µg·mL^−1^ in the respective buffers. Each condition was tested in triplicate. At specific times, 10 µL was collected and diluted 10-fold in acetonitrile before HPLC analysis (integration at λ = 254 nm). The system consisted of a Waters Separations Module 2790 coupled with a Waters 996 photodiode array detector, using a Merck Chromolith^®^ High Resolution C18, 50 × 4.6 mm reversed-phase column. A flow rate of 3 mL·min^−1^ and a gradient of (0–100) %B over 5 min were used (eluent A: water/0.1% CF_3_CO_2_H; eluent B: acetonitrile/0.1% CF_3_CO_2_H). The area ratios between the intact molecule and the degradation product peaks were determined; the calibration curve was characterized by a r^2^ = 0.9913.

The solubility of JMV5038 in the silylated vegetable oils was measured as follows: 15 mg (40.65 µmol) of JMV5038 was put into 285 mg of silylated oils. Then the mixture was gently stirred (20 rpm) for 24 h at RT, before centrifugation at 4200 rpm for 30 min. 50 µL of the supernatant was diluted 100 times in methanol for HPLC analysis. Quantitative determination was obtained by means of a calibration curve; standards were diluted in methanol within a concentration range of 0 up to 1000 µM (or 0–369 µg·mL^−1^).

### 2.4. Synthesis of Silylated Triglyceride and Monoglyceride Derivative of Castor Oil (ICOe, IGRe)

**Scheme 1:**
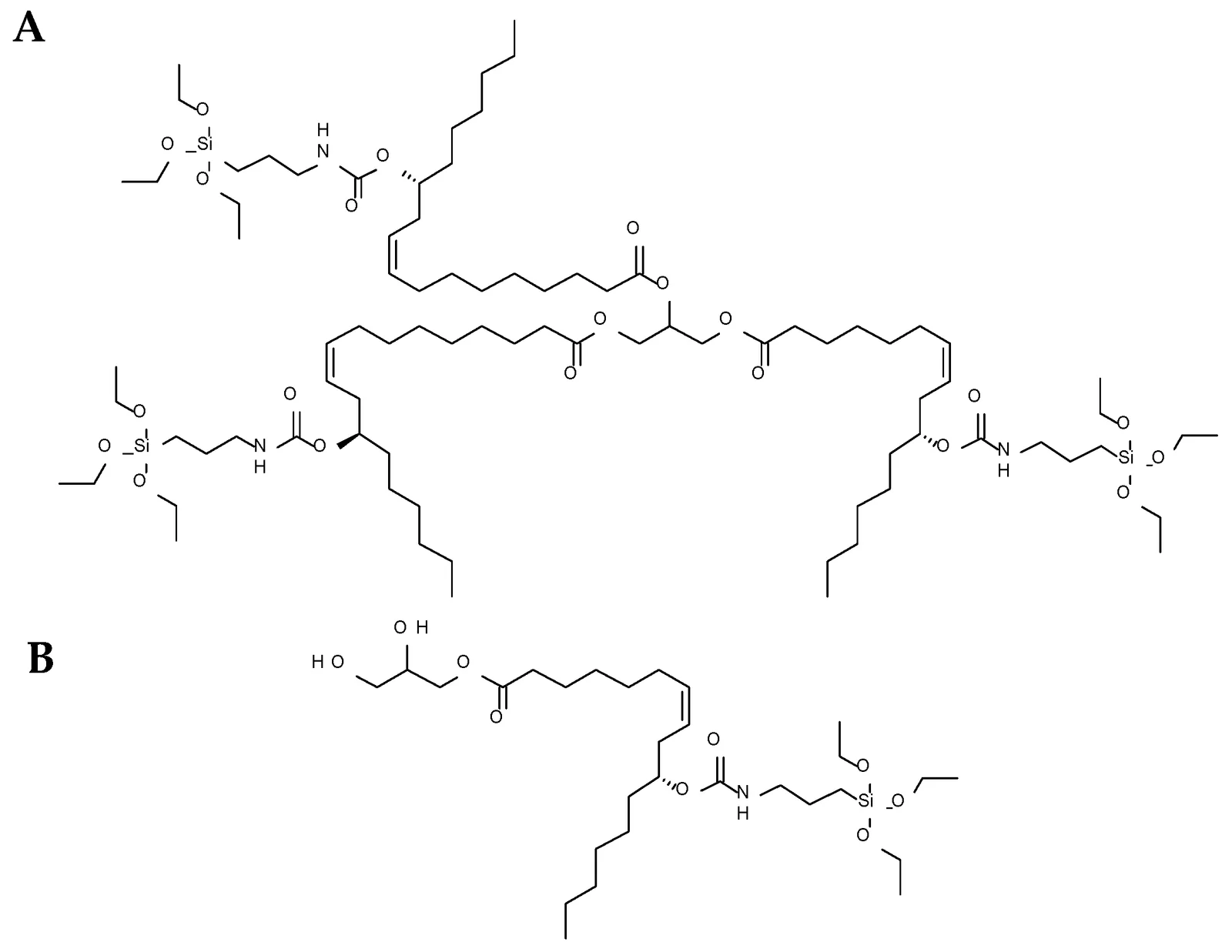
Chemical structures of (**A**) ICOe; (**B**) IGRe.

The vegetable oils (castor oil for ICOe, glyceryl monoricinoleate for IGRe, Scheme 1) were functionalized with IPTES at 60 °C for 72 h for ICOe and 22 h for IGRe, in a solvent- and catalyst-free process under an inert atmosphere. Multiple ratios of silylation were tested (1.0, 0.8, 0.6, 0.4), corresponding to the percentage of hydroxyl groups related to the isocyanate group of IPTES (i.e., 1.0 = 100% of the hydroxyl groups were silylated). The silylation reaction was monitored with IR-ATR spectroscopy (Thermo Scientific Nicolet iS50 FT-IR, Waltham, MA, USA) through the disappearance of the characteristic isocyanate band (2271 cm^−1^) and the formation of urethane bonds (1720, 1528 and 3360 cm^−1^). After the complete disappearance of the isocyanate band, the mixture was allowed to cool down to room temperature and stored under an inert atmosphere.

### 2.5. Synthesis of Silylated Conjugate Castor Oil-Lactic Acid (ICOLAe)

The conjugate castor oil-lactic acid (COLA) was synthesized following Uchida and Kouzai’s procedure [34]. A typical process is described as follows: castor oil (1 mmol) was reacted with D,L-lactide (20 mmol) for 5 h at 140 °C in the presence of SnOct as a catalyst (0.05 mmol) under an argon atmosphere.

Then, COLA (1 mmol) was functionalized with IPTES (3 mmol) in chloroform at 60 °C for 24 h with SnOct as a catalyst (0.05 mmol). The silylation was monitored with IR-ATR spectroscopy (Thermo Scientific Nicolet iS50 FT-IR), following the disappearance of the characteristic isocyanate band (2271 cm^−1^) and the formation of urethane bonds (1720, 1528 and 3360 cm^−1^). After the complete disappearance of the isocyanate band, chloroform was evaporated, and the resulting oil was stored under an inert atmosphere at RT. ^1^H NMR analysis agreed with the published data [34].

### 2.6. Viscosities of the Synthesized Oils

The viscosities of the modified vegetable oils were determined using an A&D vibroviscometer SV-10 Series (Abingdon, UK) equipped with a Julabo BC4 heating circulator set at 25 °C.

### 2.7. Formulation of Unloaded and JMV5038-Loaded Hybrid Microparticles (HMP)

Unloaded and JMV5038-loaded HMP were formulated following a thermostabilized oil-in-water emulsification process combined with sol–gel crosslinking. The outlines of the formulation method were described in a previous study [12], but have been adapted to preserve the integrity of the pH-sensitive JMV5038 by the use of a citrate buffer in the carrageenan solution (pH 3.8) [13]. Moreover, an additional solvent, ethyl acetate, has been used to solubilize conjugated castor oil (ICOLAe).

Briefly, an oil/water emulsion was prepared by mechanical stirring at 9000 rpm and 60 °C. For drug-loaded particles, JMV5038 was first solubilized in the oil phase containing SnOct (1%) prior to emulsification. The aqueous phase consisted of a thermosensitive gelling agent (κ-carrageenan at 0.5% (*w*/*w*) in distilled water), which remains liquid at 60 °C and forms a gel at RT. After the formation of the emulsion, gelation was induced by a thermal shift using an ice bath. At this stage, the emulsion was stabilized, allowing the oil droplets to remain isolated during sol–gel cross-linking, which led to particle formation over a period ranging from several days to up to three weeks. Once HMPs were formed, the gel was reheated to 60 °C to disrupt it. The particles were then washed twice with distilled water and once with ethanol by centrifugation (Fisherbrand GT2 Expert, 4200 rpm, 6 min, Thermo Fisher Scientific, Waltham, MA, USA), before freeze-drying.

The preparation efficiency of the process was calculated following Equation (2),

(2) $$Preparation\;efficiency=\frac{m_{HMP}-m_{EtOH\;loss}}{m_{VOinitial}}$$

where m_HMP_ is the final mass of hybrid microparticles, and m_EtOH loss_ is the total ethanol mass loss taken into account after crosslinking (loss of three ethanol groups per triglyceride when ICOe or ICOLAe is crosslinked, loss of one ethanol group per monoglyceride when IGRe is crosslinked).

### 2.8. Morpholgy and Size of Hybrid Microparticles

The morphology of the HMP was assessed by Scanning Electron Microscopy (SEM, Hitachi S4800 high resolution, Hitachi, Tokyo, Japan). SEM coupled with Energy Dispersive X-Ray (EDX) analysis was used to evaluate the distribution and quantify the chemical elements within the microparticles (silicon used in the cross-linking process and tin from the catalyst).

The particle size distribution was determined by laser diffraction with a Hydro 3000SM (Malvern Panalytical, Worcestershire, UK) dispersion unit with absolute ethanol as the dispersion medium. The span value (distribution width) is calculated using Formula (3).

(3) $$Span=\frac{D\left(90\right)-D(10)}{D(50)}$$

### 2.9. Thermal Analysis

TGA measurements of HMP were performed using a TGA Q50 (TA Instruments, Guyancourt, France) device under a nitrogen flow (40 mL·min^−1^). Samples weighing from 5 to 10 mg were placed in an aluminum crucible and heated from RT to 580 °C at a rate of 20 °C·min^−1^.

### 2.10. Cross-Linking Quality and Condensation Degree—Solid-State ^29^Si NMR

Solid-state ^29^Si NMR was performed in order to quantify the degree of condensation after the sol–gel cross-linking process, using two sequences of NMR at Magic Angle Spinning (MAS): Cross Polarization (CP-MAS) and a One Pulse sequence (OP-MAS) with ^1^H decoupling. Measurements were performed on a Varian VNMRS 400 MHz NMR spectrometer (Plessis-Robinson, France) equipped with a 7.5 mm Varian T3 HX MAS probe spinning at 5 kHz. CP-MAS experiments were performed with a 1.5 ms contact time and a recycle delay of 5 s, enabling identification of the different silicon environments (T^n^ units) in each sample (Table 1). For quantitative analysis, OP-MAS experiments were carried out using a 2 μs 30° pulse and a recycle delay of 60 s. Dmfit program was used to fit peaks and signal areas were integrated for each unit and expressed in percent.

To quantify the sol–gel cross-linking, condensation yields (CY) were calculated using Equation (4), taking into account the non-cross-linked oil (liquid peak, LP).

(4) CY=100%−LP

The condensation degree (CD) was calculated using Equation (5).

(5) $$CD=\frac{T^{1}+2T^{2}+3T^{3}}{3\times CY}$$

### 2.11. Encapsulation Efficiency

The experimental encapsulation efficiency of JMV5038 was determined by dispersing 10 mg of particles in 500 µL of methanol under stirring using a rotary shaker for 24 h. At that stage, all of the encapsulated JMV5038 was released into the solvent. Then, the samples were centrifuged for 6 min at 4200 rpm, and the supernatants were collected for quantification of the released drug concentration, using the previously described HPLC method after dilution by 100-fold in methanol.

### 2.12. In Vitro Release Tests of JMV5038-Loaded Hybrid Microparticles

Release tests of JMV5038-loaded HMP have been carried out in a PBS/octanol biphasic system respecting sink conditions. The experimental setup was composed of a dialysis tube (MWCO 12 kDa) containing 20 mg of JMV5038-loaded HMP suspension, immersed in 150 mL of PBS under magnetic stirring (pH 7.4; 37 °C; 200 rpm) as a physiological simulated phase, and 20 mL of octanol as a lipophilic reservoir phase that entraps the JMV5038 released from the HMP. Samples (200 µL) of the upper phase were collected at determined times prior to an HPLC analysis, and 200 µL of fresh octanol was added after each sampling to maintain the volume constant. Finally, a curve expressing cumulative JMV5038 released (%) as a function of time was plotted (average of 3 samples).

Release curves were fitted to kinetic models following these equations:

(6) $$Zero\;order:X=kt+X_{0},$$

(7) $$First\;order:\ln\left(1-X\right)=k+\;ln\left({1-X}_{0}\right),$$

(8) $$Higuchi:X=k\sqrt{t},$$

(9) $$Korsmeyer\text{-}Peppas:\ln\left(X\right)=n\ln\left(t\right)+\ln\left(X_{0}\right),$$

where *X* = drug release (%), *k* = drug release constant, and *t* = time (min).

### 2.13. In Vitro Activity Assays

Suspensions of HMP in culture medium (Dulbecco’s Modified Eagle Medium supplemented with 10% fetal bovine serum (Gibco, Waltham, MA, USA), 2 mM Glutamax^®^ (Gibco), 100 IU·mL^−1^ penicillin G sodium (Sigma Aldrich, St. Louis, MO, USA)) at different concentrations, ranging from 40 to 675 µg·mL^−1^, were shaken at 37 °C for 72 h. Then, the samples were centrifuged at 4200 rpm for 6 min, and their supernatants were collected.

A375 human melanoma cells were seeded at a density of 5000 cells per well in a 96-well culture dish plate containing 200 µL per well of culture medium for 24 h. Then, cells were exposed to the supernatants prepared above: substitution of the culture medium with the supernatants (150 µL).

After 72 h of incubation at 37 °C, cells were stained with new medium containing 50 mg·L^−1^ of neutral red (3-amino-7-dimethylamino-2-methylphenazine hydrochloride) at 37 °C for 2 h. The staining solution was then removed, and cells were washed with PBS. Finally, 100 µL of a solution containing ethanol, acetic acid and water (50/1/49 *v*/*v*/*v*) was added, and absorbance was measured at 540 nm using the microplate reader SpectraMax^®^ i3x (Molecular Devices, San Jose, CA, USA). Cell viability was normalized to the absorbance values of control wells containing only the culture medium.

The concentrations of JMV5038 in µM in the culture medium after 72 h were estimated by derivatizing the release kinetics data for the same JMV5038-loaded microparticles in the two-phase system, after 72 h for each type of microparticles. IC_50_ was determined by fitting the dose–response curves using Origin software (originLab, Northampton, MA, USA).

### 2.14. Cytocompatibility Evaluation of Unloaded Microparticles

Cytocompatibility assays were conducted according to the ISO 10993-5:2009: Biological Evaluation of Medical Devices - Part 5: Tests for in vitro cytotoxicity,., which is an extract-based compatibility test. The NIH 3T3 cells were seeded at a density of 5000 cells per well into a 96-well culture dish plate and incubated for 24 h. Simultaneously, unloaded particles were suspended in the same culture medium at concentrations of 0.1, 1 and 10 mg·mL^−1^ and incubated for 48 h. Afterwards, the particle suspensions were centrifuged (4200 rpm, 6 min) and the resulting supernatants were collected. The cells were then exposed to 200 µL of each supernatant, while wells receiving fresh culture medium served as negative controls.

After 48 h of exposure, the viability of NIH 3T3 cells was measured using a CellTiter 96^®^ AQ cell proliferation assay (Promega, Madison, WI, USA) composed of a tetrazolium compound (3-(4,5-dimethylthiazol-2-yl)-5-(3-carboxymethoxyphenyl)-2-(4-sulfophenyl)-2H-tetrazolium, inner salt; MTS) and an electron coupling reagent (phenazine methosulfate; PMS). After 3 h of incubation, the assay plate was read at 490 nm using a microplate reader (Multiskan Go, Thermo Fisher Scientific, Waltham, MA, USA). Finally, cell viability rates were normalized to the absorbance values of the negative control cells (100% viability). Results are given as the mean ± SD of three independent experiments.

### 2.15. In Vitro Enzymatic Degradation Tests

To set up the enzymatic degradation conditions for HMP, Porcine Pancreas Lipase (PPL) and *Candida rugosa* Lipase (CRL) were initially evaluated using castor oil as a model substrate. 1 mL of castor oil was added to 2 mL of lipase solutions prepared at different concentrations in PBS (pH 8.0) in 15 mL centrifuge tubes. After 4 days of incubation at 37 °C under agitation (20 rpm), the reaction was stopped by adding 10 mL of a methanol:acetone mixture (1:1 v:v). The release of free fatty acids (FFA) was then quantified by titration with a KOH solution (0.2 M), using phenolphthalein as an indicator. The percentage of hydrolysis was calculated using Equation (10).

(10) $$\%hydrolysis=\frac{n_{FFA}\times 100}{3}=\frac{c_{KOH}\times V_{eq}\times 100}{3}$$

After selecting the most active lipase for castor oil degradation, 2 mL of the corresponding lipase solution in PBS (300 units/mL, pH 8.0) was added to 20 mg of hybrid microparticles in 15 mL centrifugation tubes, before incubation at 37 °C under agitation (20 rpm). To maintain enzymatic activity, the lipase solution was renewed every four days: tubes were centrifuged (6 min, 4200 rpm), supernatants discarded and replaced with fresh enzyme solution. At the end of the study, the samples were centrifuged, supernatants discarded, and the remaining particles were washed twice with distilled water and once with ethanol (centrifugation for 6 min, 4200 rpm, for each wash). The resulting samples were freeze-dried, and the percentage of hydrolysis was determined based on the weight loss of the microparticles by Equation (11) (w_0_: initial weight; w_f_: final weight).

(11) $$\%hydrolysis=\frac{w_{0}-w_{f}}{w_{0}}\times 100$$

### 2.16. Statistical Analysis

All experiments were conducted in three independent replicates, and data were expressed as mean ± standard deviation. Multiple-way analysis of variance (ANOVA) along with a post hoc Tukey’s test was used when comparing groups using GraphPad Prism 8, and statistical significance was defined as (ns) *p* ≥ 0.055; (*) 0.01 ≤ *p* < 0.05; (**) 0.001 ≤ *p* < 0.01; (***) 0.0001 ≤ *p* < 0.001; and (****) *p* < 0.0001.

## 3. Results

### 3.1. Optimization of Hybrid Microparticles Preparation: Stabilization of JMV5038 During Microparticle Preparation

JMV5038 was selected as a model lipophilic active compound for its therapeutic potential and challenging physicochemical properties [28,29,30]. This drug exhibited potent cytotoxic activity against several melanoma cell lines, but was found to be highly lipophilic (experimental Log *p* = 3.7 ± 0.3) and sensitive to acidic conditions, making it particularly suitable for evaluating the compatibility of the formulation process with acid-labile drugs. Indeed, exposure of JMV5038 to acidic pH at high temperatures resulted in the formation of a non-cytotoxic Open-Ring Derivative (ORD) [13]. Therefore, the HMP formulation process needed to be specifically adapted in order to preserve JMV5038 integrity, while controlling cross-linking kinetics. In sol–gel chemistry, an acidic pH is known to catalyze the hydrolysis of alkoxysilanes into silanols [35,36], a step necessary for particle formation. An initial formulation protocol using an acetic acid buffer was developed; however, partial degradation of JMV5038 into ORD was observed after encapsulation. Attempts at cross-linking under neutral pH conditions were performed in order to avoid this degradation. However, the estimated gelation time under such conditions exceeded one month, rendering the process impractical. Thus, neutral pH formulations were not pursued further, and alternative acidic buffers were investigated.

**Figure 1:**
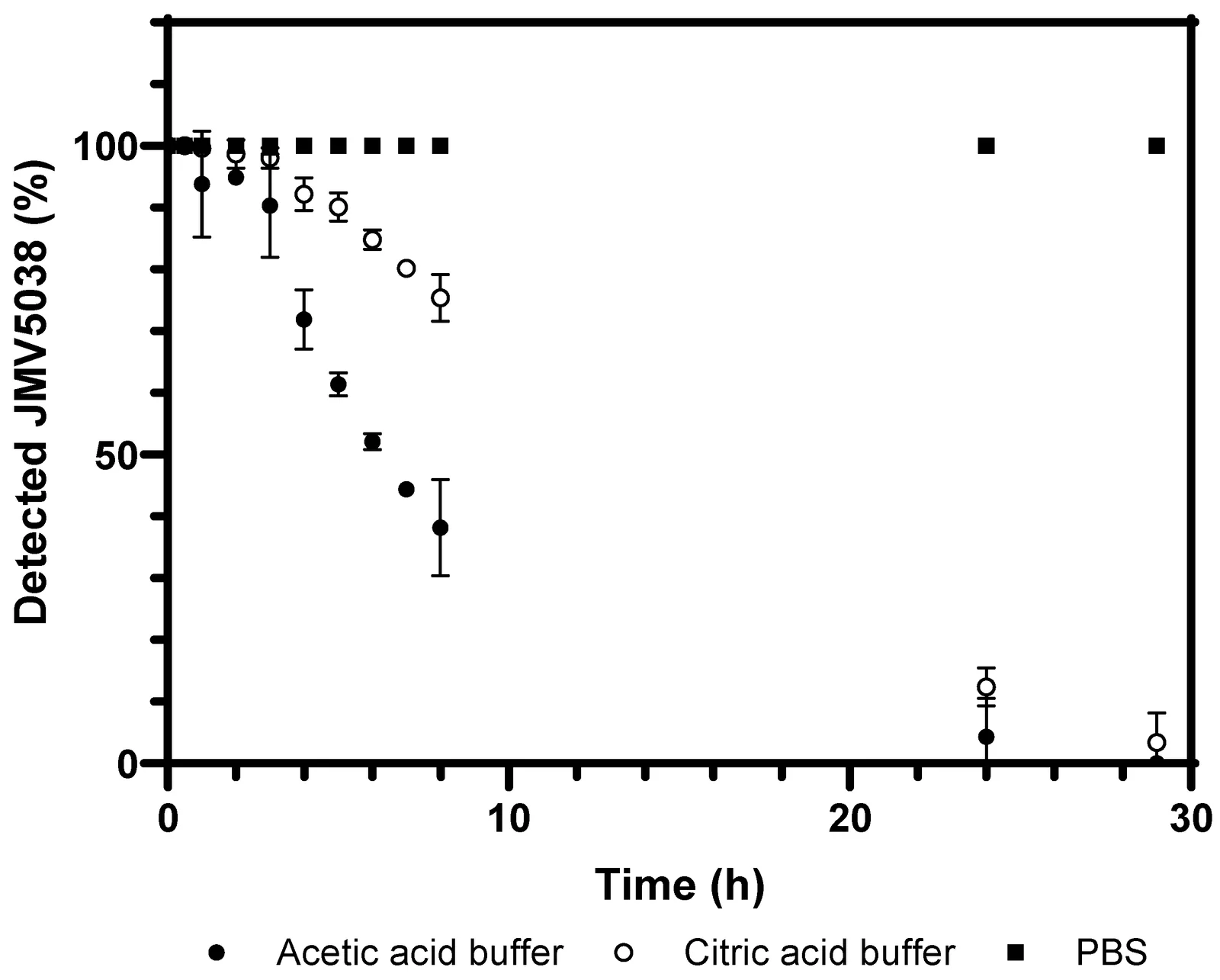
Stability study of JMV5038 in PBS (pH = 7.4), citric acid buffer (0.1 M) and acetic acid buffer (0.2 M) (pH = 3.8) at 60 °C. Data are presented as the mean +/− SD (n = 3).

To select the appropriate buffer for microparticle formulation, a stability study of JMV5038 was conducted to assess its degradation profile in acetate buffer (standard) and citrate buffer (pH 3.8) at 60 °C (formulation process temperature) (Figure 1).

JMV5038 was demonstrated to be thermally stable, as no degradation was observed in PBS at 60 °C. However, an acidic pH clearly catalyzed its degradation. The hydrolysis kinetics were different between the two acidic buffers at the same pH: degradation was slower when the citric acid buffer was used. For both cases, first traces of ORD were detected after one hour, in markedly different proportions: 6.2% of ORD in acetate buffer vs. only 0.4% in citrate buffer. Furthermore, after 5 h, less than 10% of ORD in citrate buffer vs. 38.6% of ORD in acetate buffer were detected. These results showed that a citric acid buffer is more compatible with the formulation process by significantly limiting the acid-catalyzed degradation of JMV5038.

The cross-linking quality of the HMP produced in both buffers was compared using solid-state ^29^Si NMR (Table 2). No significant difference in cross-linking quality was observed between the two acidic buffers, as both formulations exhibited similar condensation rate and comparable overall condensation degree (83% in acetic acid, 84% in citric acid). The cross-linking process was identical, requiring three weeks for both buffers.

### 3.2. Enabling Microparticle Biodegradation: Synthesis of New Modified Vegetable Oils

To enable HMP degradation, new silylated vegetable oils from glyceryl monoricinoleate were synthesized. Since this monoglyceride contains only one free hydroxyl along its fatty acid chain available to react with IPTES, each molecule bears only one trialkoxysilane moiety that can take part in the sol–gel process. Consequently, the particles formed with this oil are expected to be three times less cross-linked than those based on triglycerides described previously, potentially facilitating their degradability.

Glyceryl monoricinoleate was silylated at different rates, from 0.2 to 1.0 (IGRe). The reaction was monitored by IR, and the complete disappearance of the characteristic isocyanate band at 2271 cm^−1^ was observed after 22 h, indicating completion of the reaction, as well as the formation of urethane bonds (1720, 1528 and 3360 cm^−1^) (Figure S1, preparation of IGRe (1.0)). Their viscosities were close to 780 mPa·s (Table 3). Only two silylation rates of IGRe are reported in this paper (0.8 and 1.0), as lower rates proved insufficient to produce well cross-linked particles.

**Scheme 2:**
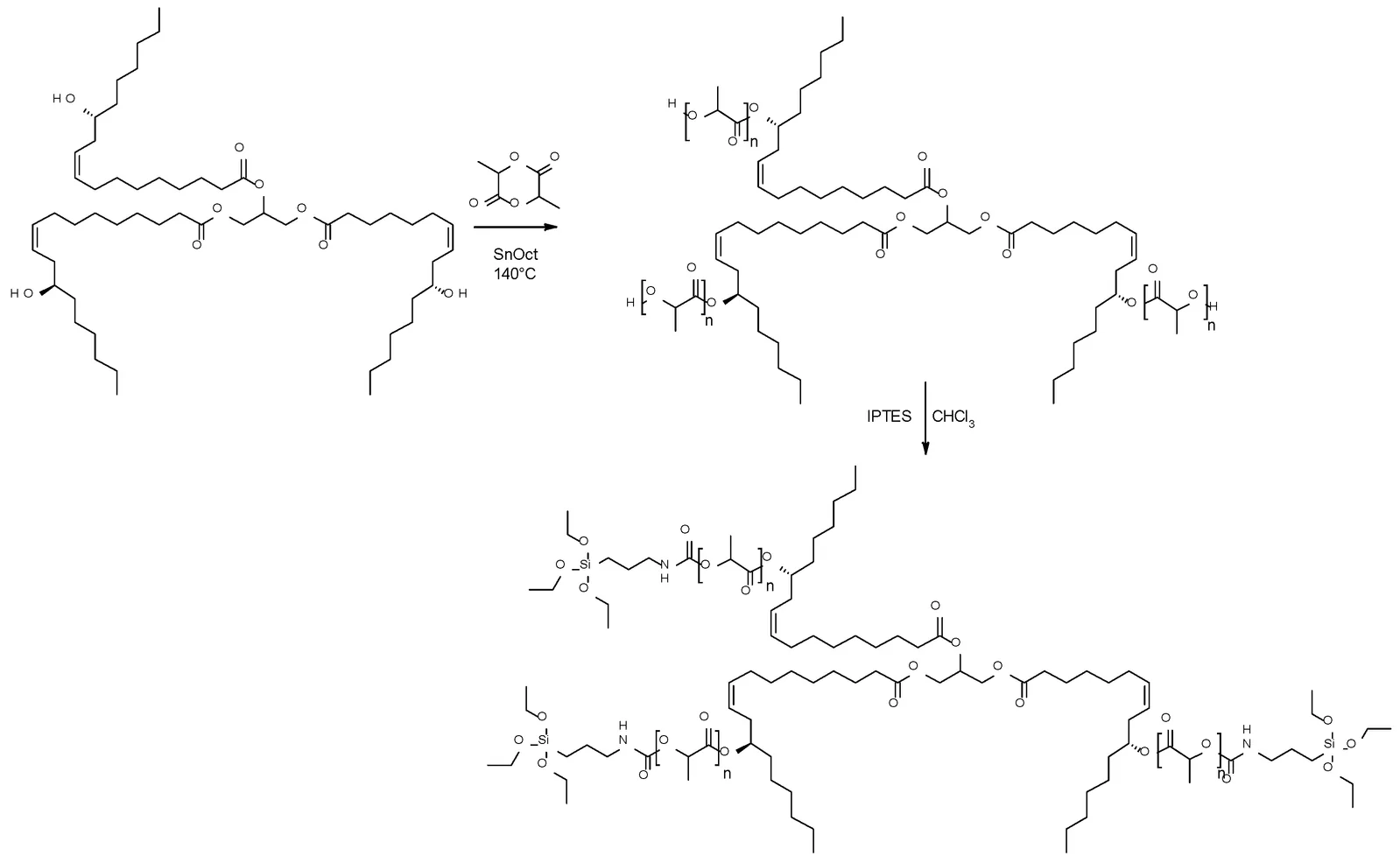
Synthesis of ICOLAe from castor oil.

A second approach to promote degradability was to synthesize a lactic acid/castor oil conjugate (ICOLAe) (Scheme 2), as lactic acid chains are known to be biodegradable [37].

The reaction involved the ring-opening polymerization of cyclic D,L-lactide onto the hydroxyl groups of castor oil, carried out at 140 °C for 5 h in the presence of SnOct [34]. ^1^H NMR studies showed the disappearance of the hydroxyl proton signals and the appearance of signals corresponding to the lactic acid moieties, including the methyl groups between 1.4 and 1.6 ppm and the C-H proton of the lactic acid moieties at 5.2 ppm. These results confirmed the formation of COLA and indicated the incorporation of 40 lactic acid moieties per triglyceride (Figure S2). The methyl groups at the end of the fatty acid tails (0.87 ppm) were used as a reference, corresponding to three protons. Moreover, IR studies showed an increase in the carbonyl band intensity at 1745 cm^−1^, due to the incorporation of lactic acid moieties, as well as enhanced C-H stretching bands between 1000 and 1400 cm^−1^, compared to unmodified castor oil (Figure S3).

The silylation of the conjugate was followed by IR and was shown to be complete after 24 h, as evidenced by the complete consumption of IPTES (disappearance of the isocyanate band at 2271 cm^−1^) (Figure S4). The presence of an intense peak at 747 cm^−1^ corresponds to the C-H band of chloroform used in the silylation reaction. It should be noted that only one silylation rate of ICOLAe is described here (1.0). Lower rates were tested but proved insufficient to achieve complete cross-linking and to produce solid microparticles, probably because of a low inorganic proportion (less than 1%), which proved to be insufficient for solid microparticle production.

Due to the added PLA chains, ICOLAe exhibited high viscosity, which was difficult to measure and hindered JMV5038 solubilization (Table 3). Therefore, the formulation process was modified by adding ethyl acetate (1 mL of ethyl acetate for 1 g of vegetable oil), a low-toxicity solvent, ICH class III, reducing the viscosity to 7.2 mPa·s.

The solubility of JMV5038 in each modified vegetable oil was studied beforehand, in order to determine their compatibility for JMV5038 formulation. The molecule was found to be more soluble in the newly developed vegetable oils, which would favor efficient encapsulation. Solubility was further enhanced when ICOLAe (1.0) was diluted in ethyl acetate, reaching values above 50 mg·mL^−1^, likely due to the good solubility of JMV5038 in this organic solvent.

### 3.3. Physicochemical Characterization of Hybrid Microparticles

Spherical-shaped HMPs were obtained with sizes ranging from 55 to 111 µm (Table 4), except for the formulation using ICOLAe, which produced much smaller particles of approximately 20 µm.

**Figure 2:**
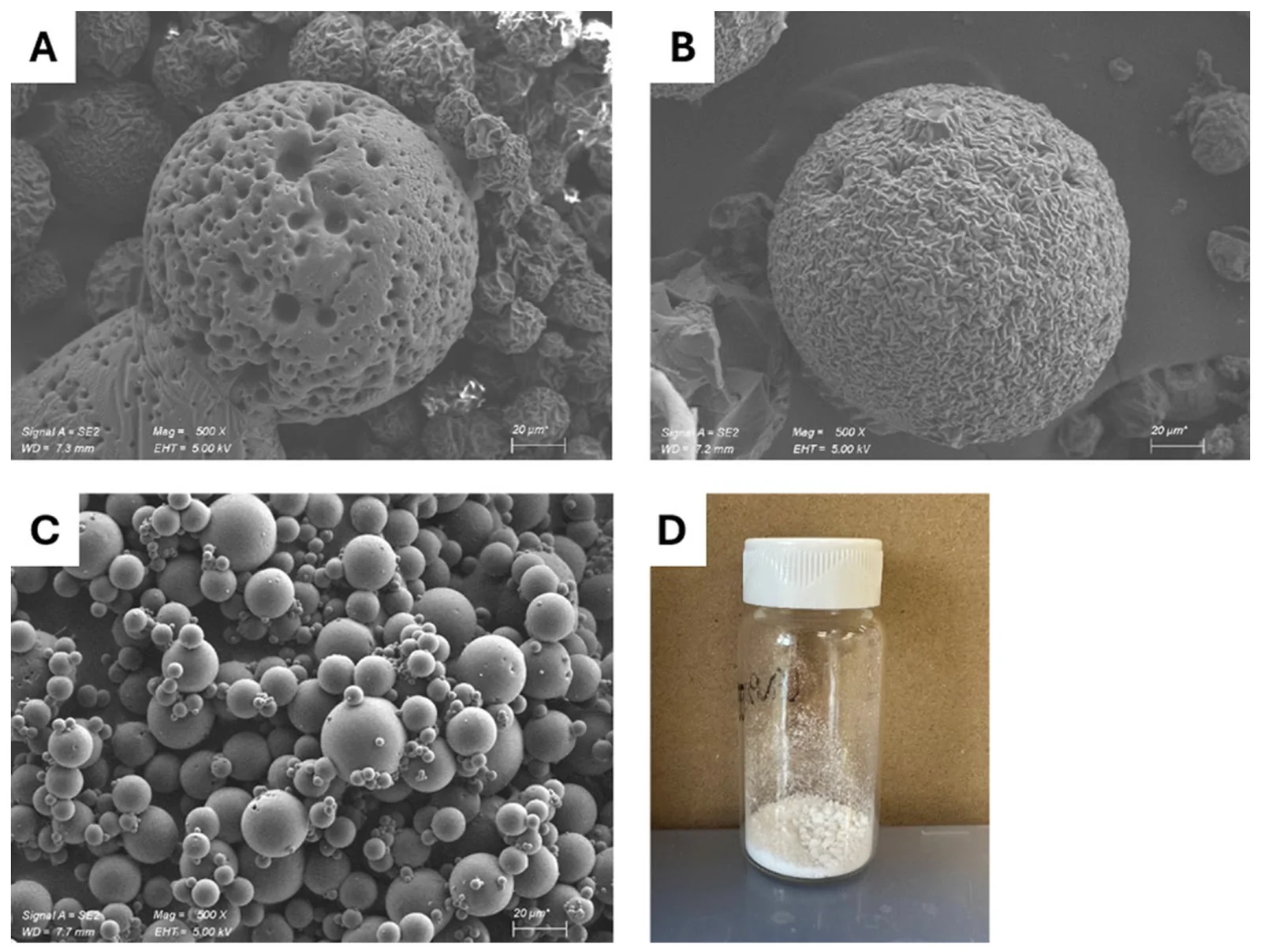
Macroscopic and microscopic pictures of HMPs: (**A**) IGRe (0.8); (**B**) IGRe (1.0); and (**C**) ICOLAe (1.0). (**D**) Macroscopic picture of ICOLAe (1.0).

The particles’ morphologies were investigated by SEM imaging (Figure 2). Particles made from monoglycerides showed a rough and irregular surface, whereas ICOLAe (1.0) particles were smooth and regular. Macroscopically, HMPs formed white powders of hard particles, as shown for those obtained with ICOLAe (Figure 2D).

**Figure 3:**
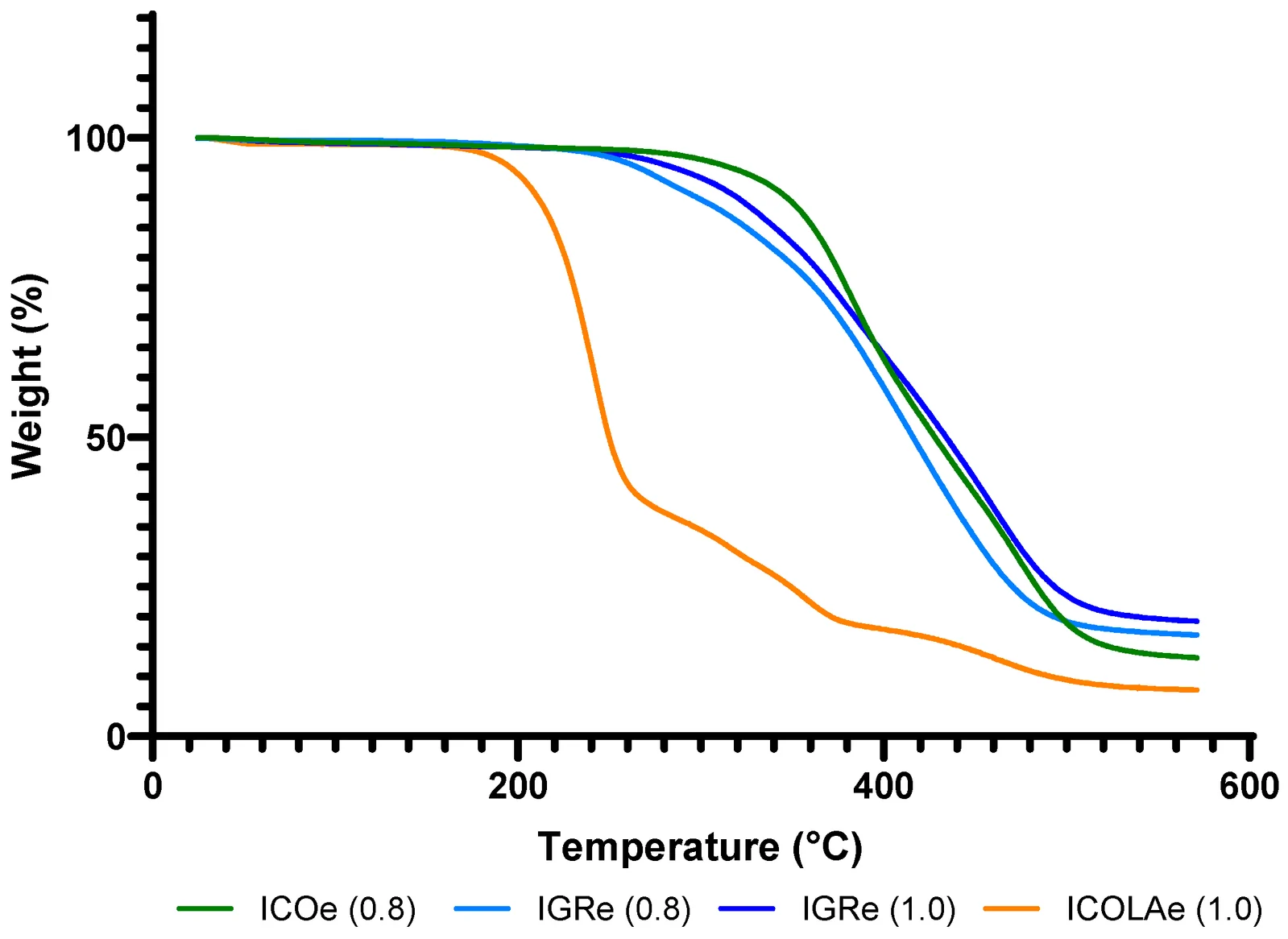
TGA curves of ICOe (0.8), IGRe (0.8), IGRe (1.0) and ICOLAe (1.0).

TGA analyses confirmed that all HMPs were of organic/inorganic hybrid nature, with approximately 15% residual mineral matter, except for ICOLAe (1.0) particles, which exhibited a lower proportion, with a significant weight loss around 200 °C attributed to the degradation of PLA chains (Figure 3).

EDX analyses of the HMP surfaces showed that the atomic compositions of multiple particles from the same batch were similar, highlighting the homogeneity of the formulation. These analyses also confirmed the hybrid composition of the microparticles, composed of both organic and inorganic matter, with silicon atomic content ranging from 1.5% to 5%, depending on the modified vegetable oil used in the formulation. Tin residues are detected at concentrations of less than 0.55% for ICOLAe microparticles.

The quality of cross-linking was assessed by solid-state ^29^Si NMR, and the proportion of each type of bond was integrated and listed in Table 5. We observed different NMR profiles for each type of HMP, and the condensation degree ranged from 67 to 88%, indicating the presence of significant cross-linking. Interestingly, HMP made from the newly synthesized oils showed little to no liquid peak (LP) in NMR, unlike those made with ICOe in our previous study.

### 3.4. Production of JMV5038-Loaded Hybrid Microparticles

JMV5038 was incorporated into the formulation at a 2% (*w*/*w*) theoretical rate. Thanks to good solubilization of the drug in the silylated oils, excellent encapsulation efficiencies ranging from 85 to 100% were achieved (Table 6). Moreover, the integrity of JMV5038 was preserved under the formulation conditions, underscoring the crucial role of the acid used in the process.

### 3.5. Release Kinetics Studies

**Figure 4:**
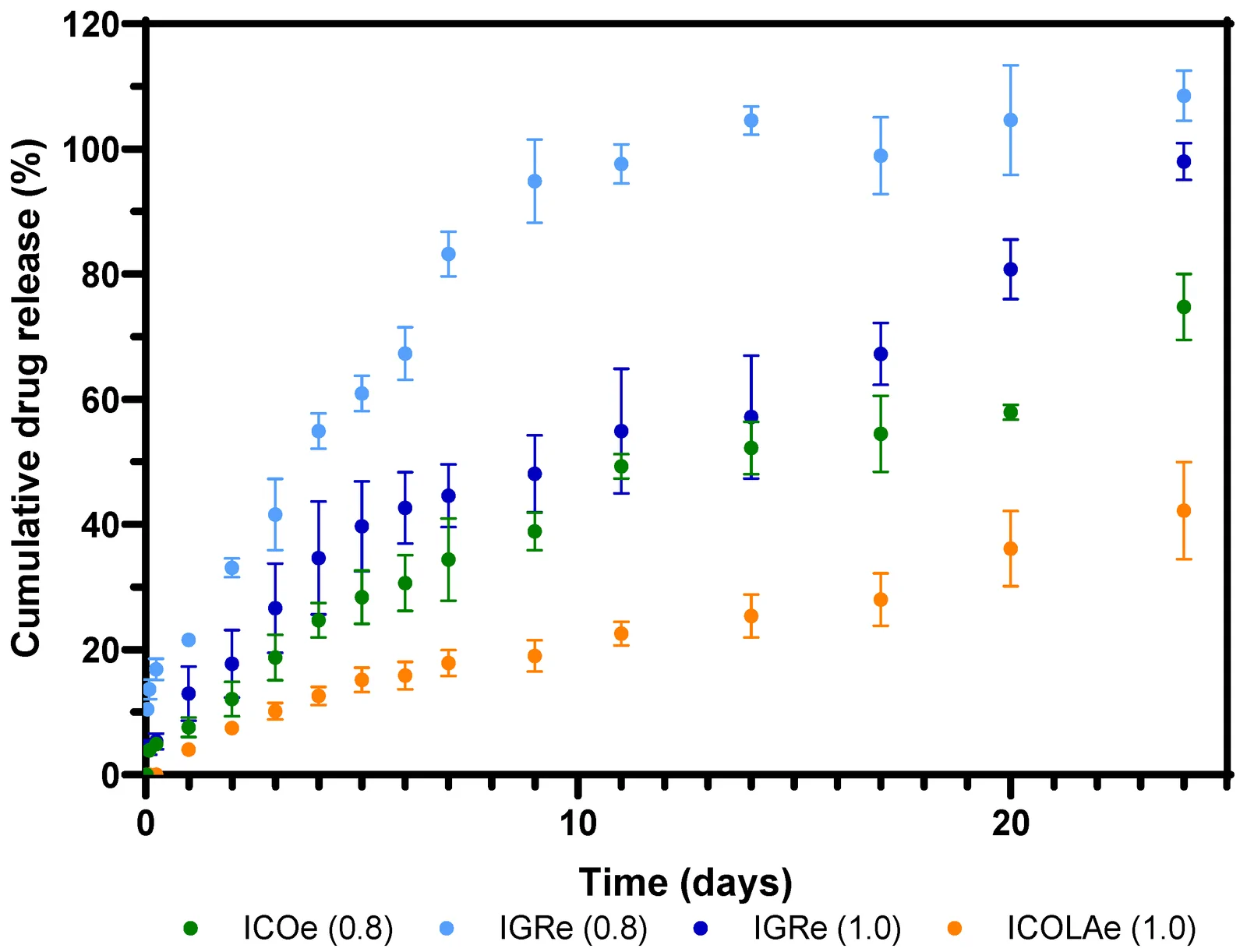
In vitro cumulative release of JMV5038 from different batches of HMP in a PBS/octanol biphasic system at 37 °C, data are presented as the mean +/− SD (n = 3).

In vitro release kinetics studies were performed on JMV5038-loaded microparticles over multiple days in a biphasic PBS/octanol model, respecting sink conditions. All microparticles showed a sustained release of the drug, even if their physical properties (size, mineral part and condensation rate) were very different (Figure 4). Particles based on IGRe (0.8) showed the fastest release profile, reaching 100% of release in 11 days, compared to only 22.5% for ICOLAe particles over the same period. IGRe (0.8) particles also showed a burst effect, releasing 21.5% of JMV5038 within the first 24 h, whereas the release remained below 10% for all other particles. It should be noted that experimental errors arising from the multiple dilution steps resulted in percentages exceeding 100%.

**Figure 5:**
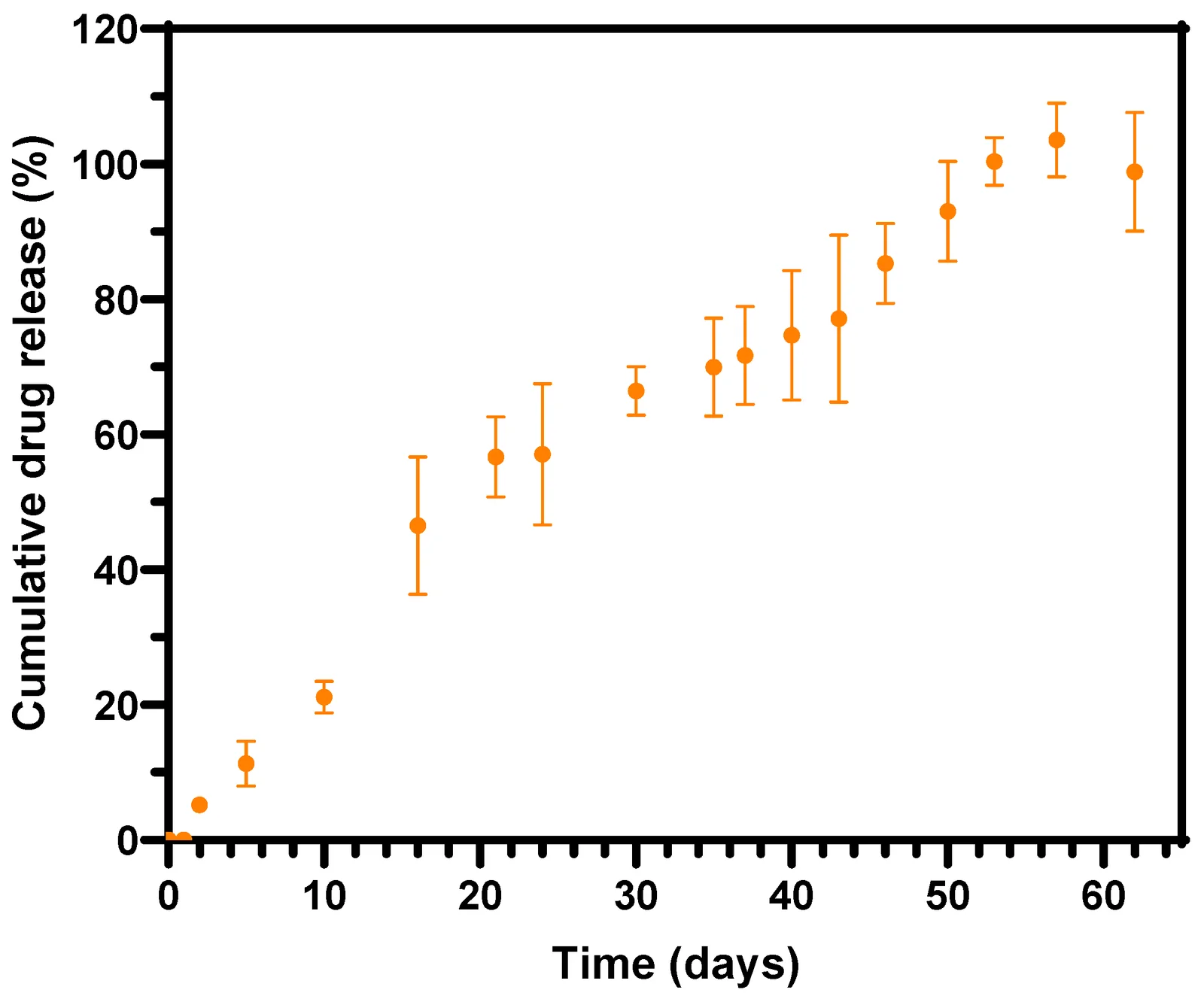
In vitro cumulative release of JMV5038 from ICOLAe (1.0) hybrid microparticles, data are presented as the mean +/− SD (n = 3).

ICOLAe-based microparticles, despite their smaller size, released the drug very slowly, reaching complete liberation after 53 days (Figure 5).

The release rates were fitted to various kinetic models (Table 7; Figure S5): all kinetics were best fitted with a Higuchi-type release (highest R^2^), meaning that the rate of release was slow and constant. Following the Korsmeyer–Peppas equation, IGRe (0.8) showed n < 0.45; thus, the mechanism of release was governed by diffusion (Fickian diffusion), whereas other formulations had 0.45 < n < 0.89; thus, a combination of diffusion and polymer relaxation and/or swelling had a role in drug release.

### 3.6. Cytocompatibility Assays

**Figure 6:**
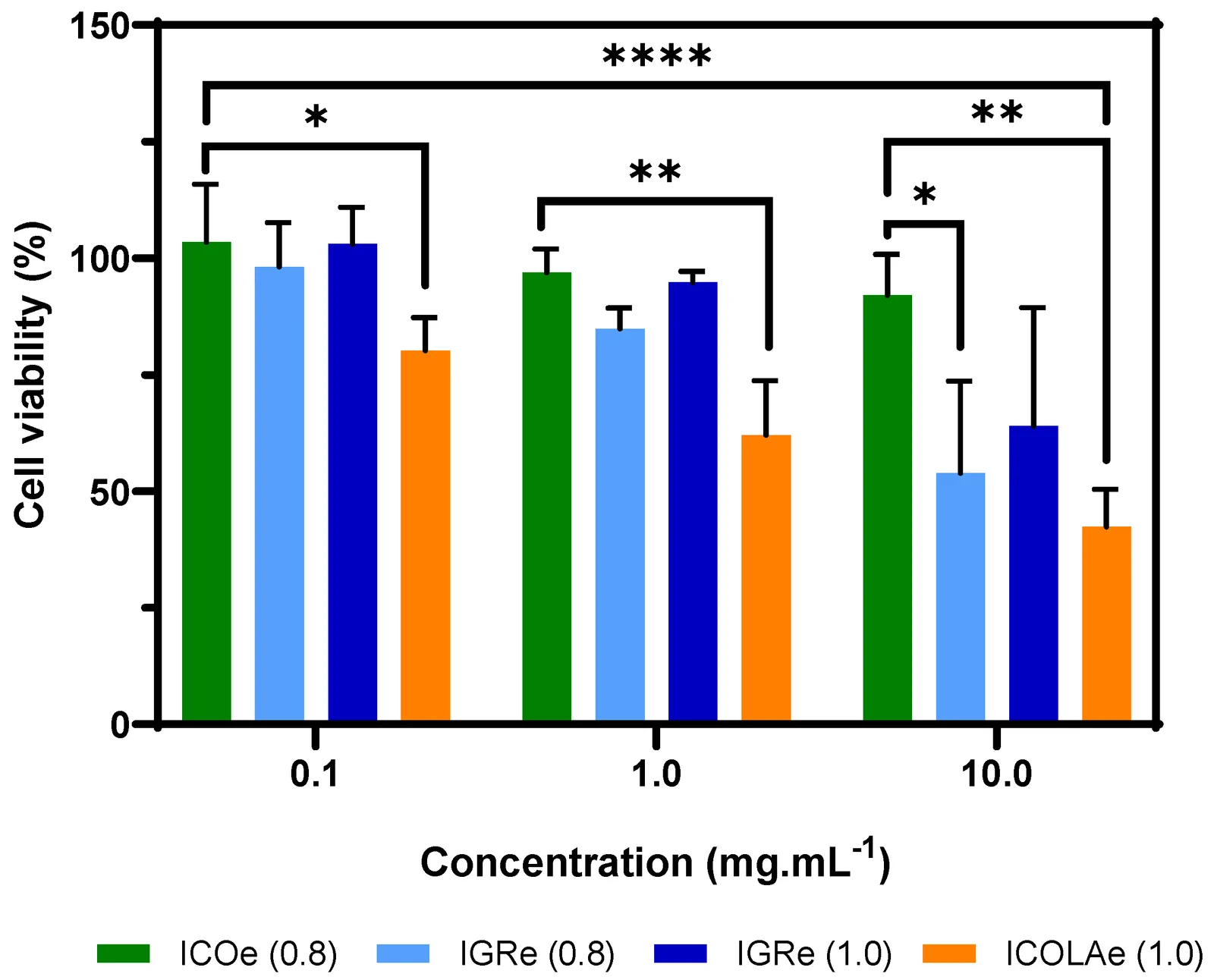
Cell viability of NIH 3T3 cells of multiple particle suspensions (formulated with SnOct, without JMV5038) at 0.1, 1 and 10 mg·mL^−1^; 48 h incubation; MTS revelation. Data are presented as the mean +/− SD (n = 3) and (*) 0.01 ≤ *p* < 0.05; (**) 0.001 ≤ *p* < 0.01; and (****) *p* < 0.0001.

The cytocompatibility of the hybrid particles without JMV5038 was assessed on NIH 3T3 fibroblast cells at three concentrations to ensure their safety for healthy tissues (Figure 6). To do so, supernatants after 48 h of HMP incubation in culture media were tested on cells.

At the lowest concentration (0.1 mg·mL^−1^), no cytotoxic effects were observed for any batch of microparticles, indicating good biocompatibility at low doses. However, with increasing concentrations, a decrease in cell viability was noted for all formulations, except ICOe. At 10 mg·mL^−1^, cell viability dropped to 54% and 64% for IGRe 0.8 and 1.0, respectively, and to 42% for ICOLAe particles. In contrast, ICOe-based microparticles maintained high cytocompatibility even at the highest tested concentration, making them the most biocompatible formulation among those tested.

### 3.7. In Vitro Activity Assays

**Figure 7:**
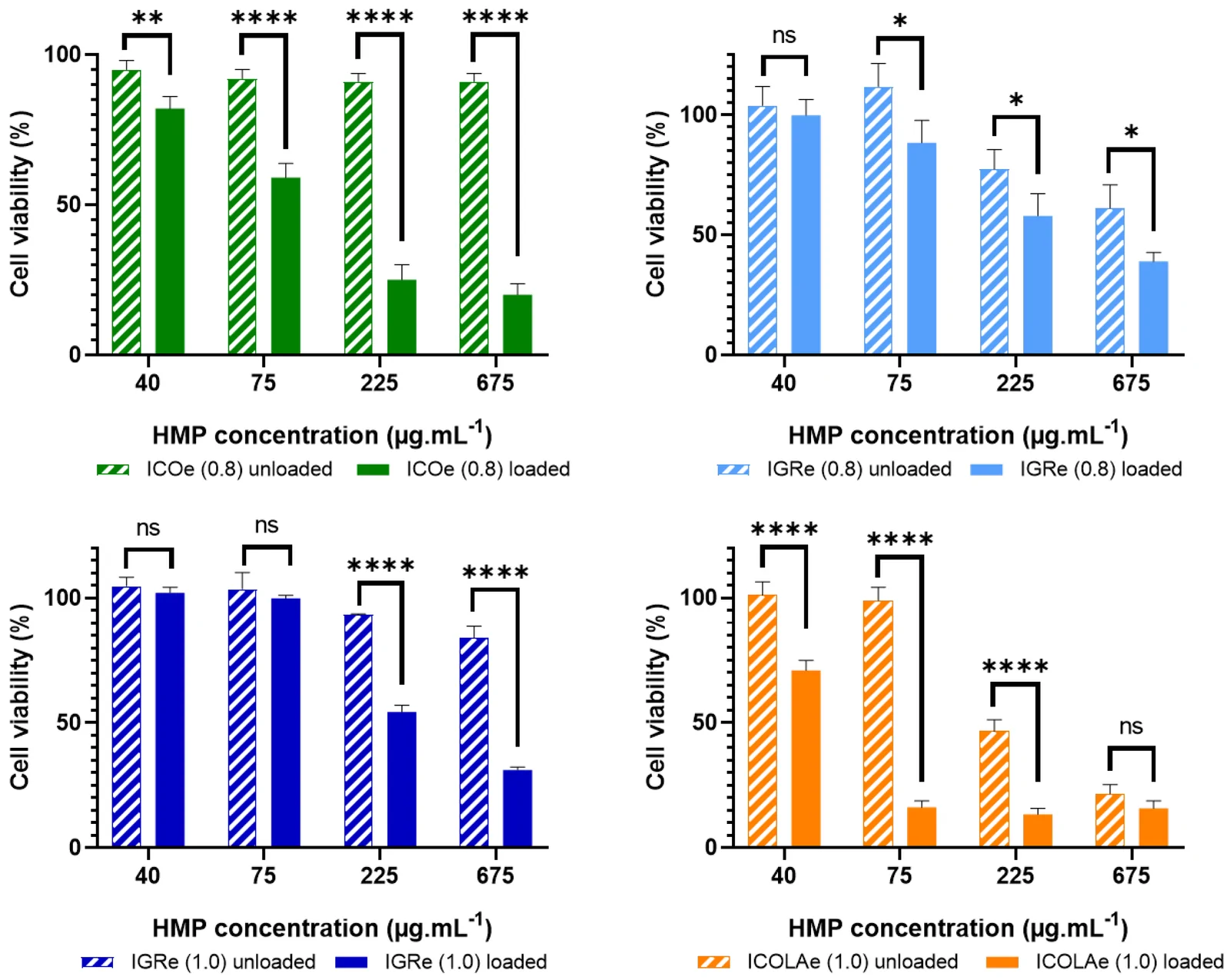
In vitro activity assays of unloaded and JMV5038-loaded hybrid microparticles on A375 melanoma cells. Data are presented as the mean +/− SD (n = 3) and (*) 0.01 ≤ *p* < 0.05; (**) 0.001 ≤ *p* < 0.01; and (****) *p* < 0.0001.

The in vitro cytotoxic activity of the hybrid microparticles was assessed on A375 melanoma cells at multiple concentrations of microparticles (40, 75, 225 and 675 µg·mL^−1^) (Figure 7).

JMV5038-loaded microparticles showed a significant cytotoxicity activity on A375 cells, with an IC_50_ of the same order of magnitude as the JMV5038 alone (IC_50_ = 2.10 ± 0.42 µM, or 0.738 µg·mL^−1^) [30], highlighting the conservation of its biological activity during its processing and in the culture medium. Indeed, for ICOe and IGRe, we can estimate that the IC_50_ is reached at microparticle concentrations around 75 and 225 µg·mL^−1^, whereas for ICOLAe at concentrations below 75 µg·mL^−1^. For ICOLAe, it should be noted that this toxicity is linked solely to JMV5038, as the microparticles alone are well tolerated at 75 µg·mL^−1^.

### 3.8. In Vitro Enzymatic Degradability Assays

The aim of this experiment was to demonstrate the degradability of microparticles under favorable conditions by using the most efficient, although non-physiological, process as proof-of-concept, using lipases, which are esterases capable of degrading oils. The enzymatic activity of two lipases towards castor oil was evaluated: porcine pancreatic lipase (PPL) and fungal lipase from *Candida rugosa* (CRL). These initial tests identified CRL as the most effective candidate for enzymatic degradation studies. Indeed, at a concentration of 300 units/mL (which corresponded to the solubility limit of the lipase in PBS (pH 8.0, 37 °C)), 69% of the castor oil was hydrolyzed after 4 days, compared to 42% when using PPL under the same conditions. It was necessary to adjust the pH of the PBS used in this study to pH 8 to counteract the acidification of the medium due to the formation of free fatty acids during hydrolysis.

**Figure 8:**
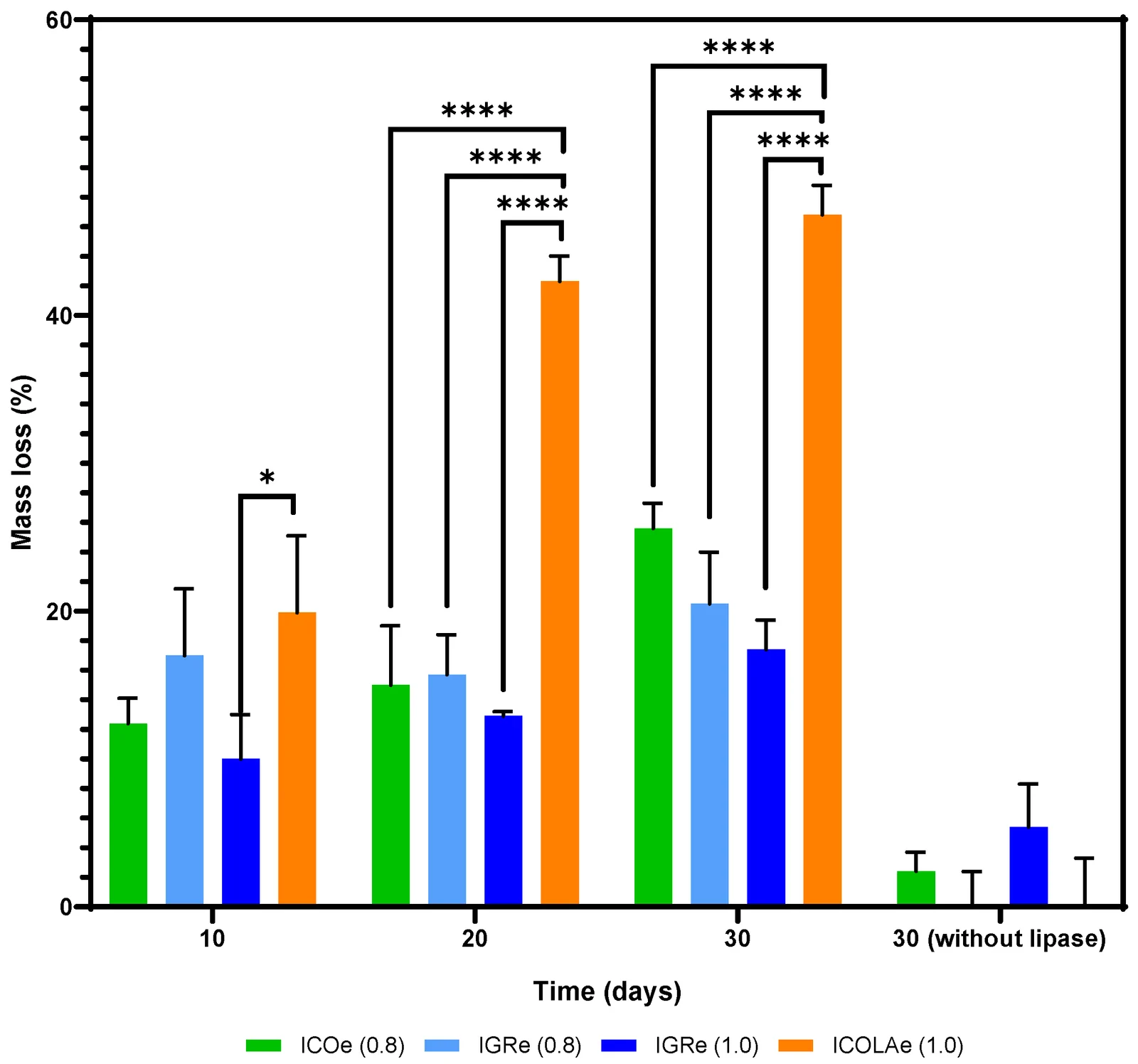
In vitro enzymatic degradability study on different batches of microparticles in *Candida rugosa* lipase solution in PBS (300 units/mL), at 37 °C, rotation at 20 rpm. Data are presented as the mean +/− SD (n = 3) and (*) 0.01 ≤ *p* < 0.05; and (****) *p* < 0.0001.

CRL was then used to evaluate the degradability of the hybrid microparticles by monitoring their weight loss after 10, 20 or 30 days of enzymatic treatment (Figure 8). All particle batches proved to be degradable in the presence of the lipase, with degradation increasing over time: 10–20% after 10 days, 12–42% after 20 days and 17–46% after 30 days. In contrast, less than 10% degradation was observed for microparticles incubated in the absence of lipase after 30 days.

No significant difference in mass loss was observed among ICOe (0.8), IGRe (0.8) and IGRe (1.0) particles, showing a similar degradation profile for these formulations until day 20. As expected, microparticles of ICOLAe showed the highest and most significant degradability rate, with more than 45% hydrolysis after 30 days of the experiment.

## 4. Discussion

Before the formulation of JMV5038 in HMP, the stability of the molecule was evaluated under the acidic conditions required for the sol–gel process. Among the investigated buffers, citrate proved to be the most suitable choice for the formulation of JMV5038, thanks to a significant reduction in the drug degradation rate, while maintaining comparable cross-linking characteristics to those obtained with the acetate buffer. While the influence of buffer composition has been extensively studied in protein stability and RNA encapsulation [38,39,40], its impact on small molecule stability remains less explored. The difference observed may be attributed to the distinct physicochemical properties of the two buffering agents: citrate, a tricarboxylic acid with multiple pKa values, presents a higher molecular charge density and the ability to form hydrogen bonding networks, which likely creates a less reactive microenvironment, decreasing the effective proton activity experienced by acid-sensitive molecules. In contrast, acetate is a smaller monocarboxylic acid with a narrower buffering range, which may allow for a higher localized proton concentration and thus faster drug degradation. These combined effects support the hypothesis that citrate is a more suitable buffer for encapsulation processes involving pH-sensitive compounds under mildly acidic conditions; further studies on degradation mechanisms across multiple buffers would be needed to confirm this. Nonetheless, the citrate buffer has been selected for the JMV5038 formulation in HMP for this study, as it resulted in a slower degradation rate compared to the acetate buffer.

To further optimize the formulation, several silylated vegetable oil derivatives (IGRe and ICOLAe) were investigated to identify matrices providing favorable drug loading and release properties. Regardless of oil composition, JMV5038 exhibited higher solubility in IGRe than in the previously described oil (ICOe), resulting in improved drug encapsulation and loading efficiency. Solubility was further enhanced when ICOLAe (1.0) was diluted in ethyl acetate, reaching values above 50 mg·mL^−1^, likely due to the good solubility of JMV5038 in this organic solvent. After cross-linking, HMP exhibited comparable sizes, ranging from 55 to 111 µm. However, ICOLAe (1.0) particles were much smaller. This reduction in size was initially attributed to the incorporation of ethyl acetate as a co-solvent, which could have modified the oil-in-water emulsion behavior. However, when ICOe (0.8) was solubilized in ethyl acetate (1 mg·mL^−1^) prior to formulation, microparticles with sizes comparable to those obtained using the original process were produced. Thus, the reduced size of ICOLAe could not be attributed to the formulation procedure alone, but is more likely related to the specific physicochemical properties of ICOLAe. The presence of PLA segments may confer surfactant-like properties to the polymer chains [41,42], promoting the formation of smaller oil droplets during emulsification and consequently smaller microparticles. Furthermore, the incorporation of PLA chains increased the overall organic fraction of the particles, resulting in a lower residual mineral content after TGA analysis.

The morphology of the particles was studied by SEM imaging. Based on their chemical structure, a rougher surface morphology was expected for monoglyceride-based particles, as they contain only one fatty acid tail, limiting the degree of cross-linking, compared to the triglyceride ICOLAe (1.0), which provides three fatty acid chains available for functionalization and participation in network formation. The cross-linking quality was assessed by solid-state ^29^Si NMR: all HMP formulations showed a higher proportion of T^2^ and T^3^, indicating good cross-linking efficiency. Interestingly, the absence of the liquid peak (LP) in NMR further supports the homogeneous and effective cross-linking process achieved with these oils. When ICOLAe was used, more than 70% of T^3^ was observed, showing a very strong cross-linking network, which could limit its degradability and slow down the release of the encapsulated compound. In contrast, IGRe (0.8) particles exhibited only 26% T^3^, meaning that the network is less cross-linked, potentially enabling the faster release profile observed in the release kinetic studies. Thus, the degree of silylation, as assessed using the castor oil monoglyceride IGRe, 0.8 or 1.0, has a significant effect on cross-linking and the resulting drug release profile. IGRe (0.8), with 26% T^3^, showed rapid drug release within 11 days, associated with an initial burst effect, whereas IGRe (1.0), with 57% T^3^, exhibited a more regular and gradual release profile, achieving complete release after 25 days, without an initial burst. It should be emphasized that the condensation degrees (67% vs. 84% for IGRe (0.8) and (1.0), respectively) also contributed to these differences in release profiles and may account, at least in part, for the sustained release observed with IGRe (1.0).

Overall, both condensation yield and condensation degree of the particles significantly influenced drug release kinetics, with lower condensation degrees and the use of monoglyceride oils favoring faster compound release. It should also be noted that another factor may contribute to the prolonged release profile: the presence of a liquid peak in the ^29^Si NMR spectra. This signal corresponds to non-cross-linked oil and may facilitate drug entrapment within the particles, thereby contributing to the prolonged release observed over 5 weeks.

After 30 or 65 days of release studies, microscopic examination of all batches revealed that all microparticles remained completely intact, indicating the absence of significant hydrolytic degradation under the tested conditions and supporting a diffusion-controlled release mechanism. This hypothesis was further supported by mathematical interpretation of the release data, which resulted in linear profiles fitting a Higuchi-type release (Table 7).

To investigate whether enzymatic degradation of hybrid microparticles could be achieved, a proof-of-concept evaluation was performed using CRL lipase. Initially, IGRe degradation was expected to be improved, thanks to lower cross-linking and the presence of free hydroxyls potentially available for enzymatic degradation processes. However, IGRe (0.8) and IGRe (1.0) particles displayed degradation profiles comparable to those of ICOe (0.8). This behavior may be attributed to the higher cross-linking quality of IGRe particles, as suggested by the disappearance of the liquid oil peak observed in solid-state NMR analysis. In contrast, the incorporation of PLA segments into the oil-based matrix significantly improved enzymatic degradability, thanks to the hydrolysis of PLA moieties. Indeed, PLA is made of polyester chains, which are readily degraded thanks to the esterase action of the lipase. Significant differences in degradability compared to all other types of particles were already evident after 20 days, highlighting the potential of PLA conjugation for promoting enzymatic degradation. In this paper, only a proof-of-concept degradation, using a fungal lipase, has been investigated for a first evaluation of degradability. Further biodegradation studies combined with in vivo efficacy studies with JMV5038-loaded microparticles will be investigated to amplify their potential biomedical application.

Then, EDX analyses suggested that residual tin is incorporated into microparticles, estimated at a tin mass percentage of 0.55 ± 0.05% per microparticle sample. This tin residual could explain the moderate cytotoxicity of the unloaded particles at higher concentrations on NIH-3T3 cells (Figure 7). SnOct is used in ICOLAe synthesis, as well as in the cross-linking reaction. This catalyst is known to be cytotoxic [43], although it is authorized for use in medical devices, provided it does not exceed 640 µg/day of tin (II) for parenteral administration [44]. In vivo biocompatibility studies will be performed at a later stage to confirm the safety of these hybrid microparticles, and future work on a process using nontoxic catalysts is underway.

Finally, by linking the release kinetic studies with in vitro activity assays on A375 melanoma cells, we were able to show the preservation of the cytotoxic activity of JMV5038, even after its encapsulation (IC_50_ = 2.10 ± 0.42 µM). Experiments are planned to evaluate, in vivo, the activity of microparticles loaded with JMV5038 administered subcutaneously to mice bearing xenograft tumors derived from A375 melanoma cells over 1 month. These experiments will aim to assess whether these hybrid microparticles can provide sustained release of the cytotoxic agent and improve its antitumor efficacy against melanoma tumors.

## 5. Conclusions

A new generation of silylated castor oil-based hybrid microparticles was developed to overcome the limited degradability of particles and harsh acidic conditions that constrained previous generations of vegetable oil-based carriers, while preserving the integrity of the acid-labile lipophilic antitumor compound JMV5038. The optimized formulations exhibited improved drug-solubilizing properties, enabling efficient encapsulation and sustained release over 1 to 2 months. In addition, the hybrid microparticles were cytocompatible at concentrations up to 0.1 mg·mL^−1^, and remained susceptible to lipase-mediated degradation, highlighting their potential as degradable long-acting drug delivery systems.

To achieve this, new modified vegetable oils were synthesized. IGRe, a silylated glyceryl monoricinoleate, improved JMV5038 solubility through its surfactant properties and IGRe (1.0) based microparticles ensured complete release within 25 days. Similarly, ICOLAe, associating PLA chains onto castor oil, enhanced the solubilizing efficiency of the microparticles while extending release up to 53 days. Both derivatives conferred enzymatic degradability, with hybrid microparticles reaching up to 46% weight loss after 30 days in the presence of lipases.

Altogether, these results position vegetable oil-based hybrid microparticles as a versatile and degradable platform for the encapsulation and prolonged release of lipophilic, acid-sensitive antitumor compounds, paving the way for their potential use as subcutaneous implants, after further in vivo compatibility and biodegradability assays.

## Figures and Tables

**Table 1:** Types of silicon bonds in sol–gel cross-linked materials and their chemical shifts.

Names	T^0^	T^1^	T^2^	T^3^
Structures	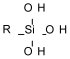	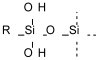	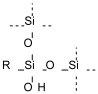	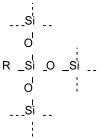
Chemical shifts (ppm)	−48	−53	−57	−67

**Table 2:** Evaluation of the quality of cross-linking by solid-state ^29^Si NMR, between microparticles formulated in acetate or citrate media.

Buffer	Acetate Buffer	Citrate Buffer
T^0^	4%	2%
T^1^	3%	2%
T^2^	34%	39%
T^3^	59%	57%
Condensation degree	83%	84%

**Table 3:** Physicochemical properties of the different modified vegetable oils at 25 °C.

Modified Oils	Molecular Weight (g/mol)	Viscosity (mPa·s)	Density	Solubility of JMV5038 (mg·mL^−1^)
ICOe (0.8)	1467.5	764	0.994	2.3 ± 0.6
IGRe (0.8)	570.4	780	0.965	23.6 ± 0.5
IGRe (1.0)	619.9	775	1.005	10.7 ± 2.8
ICOLAe (1.0)	4558.6	N.D. ^1^	N.D. ^1^	N.D. ^1^
ICOLAe (1.0) + ethyl acetate	4558.6	7.2	N.D.^1^	>50

^1^ N.D. = not determined.

**Table 4:** Size and size distribution (span) of hybrid microparticles (n = 3).

Modified Oil	ICOe (0.8)	IGRe (0.8)	IGRe (1.0)	ICOLAe (1.0)
Mean size (µm)	55 ± 4	111 ± 1	74 ± 1	20 ± 0
Span	2.03 ± 0.03	1.25 ± 0.02	1.40 ± 0.06	1.40 ± 0.00

**Table 5:** Solid-state ^29^Si NMR integrations of sol–gel condensation peaks of unloaded HNPs.

Modified Oil	ICOe (0.8)	IGRe (0.8)	IGRe (1.0)	ICOLAe (1.0)
LP	17%	4%	0%	0%
T^0^	4%	10%	1%	1%
T^1^	6%	5%	2%	6%
T^2^	28%	55%	40%	20%
T^3^	45%	26%	57%	73%
Condensation degree	71%	67%	84%	88%

**Table 6:** Preparation characteristics of JMV5038-loaded hybrid microparticles.

Modified Oil	ICOe (0.8)	IGRe (0.8)	IGRe (1.0)	ICOLAe (1.0)
Encapsulation rate	1.7%	2%	2%	2%
Encapsulation efficiency	85%	100%	100%	100%
Preparation efficiency	85%	72%	78%	65%

**Table 7:** Kinetic model interpretation values for the drug release studies.

Formulation	Zero-Order (R^2^)	First-Order (R^2^)	Higuchi (R^2^)	Korsmeyer–Peppas (n)
**ICOe (0.8)**	0.956	0.967	0.990	0.607
**IGRe (0.8)**	0.963	0.909	0.990	0.404
**IGRe (1.0)**	0.954	0.919	0.989	0.568
**ICOLAe (1.0)**	0.973	0.975	0.979	0.681

## Data Availability

The original contributions presented in this study are included in the article/Supplementary Materials. Further inquiries can be directed to the corresponding author.

## Supplementary Materials

The following supporting information can be downloaded at https://www.mdpi.com/article/10.3390/pharmaceutics18091145/s1. Figure S1: Infrared spectra monitoring of the glyceryl monoricinoleate silylation (IGRe (1.0) synthesis). Time = 0 h (purple), 1 h (light green), 2 h (light blue), 3 h (pink), 4 h (dark blue), 6 h (dark green), 22 h (red). Figure S2: ^1^H NMR spectrum of COLA. (400 MHz, CDCl_3_). Figure S3: Infrared spectra of COLA (red) compared to castor oil (blue); Figure S4: Infrared spectra for the monitoring of COLA silylation. Time = 0 h (blue), 4 h (purple), 7 h (orange), 24 h (red). Figure S5: Release kinetics models of in vitro JMV5038 release tests.

## Abbreviations

The following abbreviations are used in this manuscript:

HMP	Hybrid microparticle
PLA	Polylactic acid
PLGA	Poly(lactic-co-glycolic) acid
ICOe	Ethoxysilylated castor oil
IPTES	Isocyanatopropyl triethoxysilane
SnOct	Tin(II) 2-ethylhexanoate
PPL	Porcine Pancreas Lipase
CRL	*Candida rugosa* Lipase
MTS	Mitochondrial activity assay
DMEM	Dulbecco’s Modified Eagle Medium
MW	Molecular Weight
OECD	Organization for Economic Co-operation and Development
HPLC	High-Performance Liquid Chromatography
IGRe	Ethoxysilylated monoglyceryl ricinoleate
IR	Infrared
IR-ATR	Infrared-Attenuated Total Reflectance
RT	Room Temperature
ICOLAe	Ethoxysilylated conjugate Castor Oil-Lactic Acid
COLA	Conjugate Castor Oil-Lactic Acid
SEM	Scanning Electron Microscopy
EDX	Energy-Dispersive X-ray
TGA	Thermogravimetric Analysis
MAS	Magic Angle Spinning
CP-MAS	Cross Polarization-Magic Angle Spinning
OP-MAS	One Pulse-Magic Angle Spinning
CY	Condensation Yield
CD	Condensation Degree
LP	Liquid Peak
PBS	Phosphate-Buffered Saline
MWCO	Molecular Weight Cut-Off
PMS	Phenazine methosulfate
FFA	Free Fatty Acid
ORD	Open Ring Derivative
ND	Non Determined
