# Supplementary material for: Development of Degradable Silylated Castor Oil-Based Microparticles for the Sustained Release of a Lipophilic Anti-Cancer Drug
Source: Pharmaceutics. 2026 Sep 11;18(9):1145. doi: 10.3390/pharmaceutics18091145 (PMC13610840; doi:10.3390/pharmaceutics18091145)
Supplement: Supplementary file 1 [file pharmaceutics-18-01145-s001.zip › pharmaceutics-4485603-supplementary.pdf]

Development of Degradable Silylated Castor Oil-Based Microparticles for the Sustained Release of a Lipophilic Anti-Cancer Drug

Lucas M. Favre,[1,2] Ezechiel Romone,[1] Pierre Cuq,[1] Nicolas Masurier,[1] Anne Aubert-Pouëssel[2*]

[1] Institut des Biomolécules Max Mousseron (IBMM), Univ Montpellier, CNRS, ENSCM, Montpellier, France

[2] Institut Charles Gerhardt Montpellier (ICGM), Univ Montpellier, CNRS, ENSCM, Montpellier, France

* Correspondence: anne.aubert@umontpellier.fr

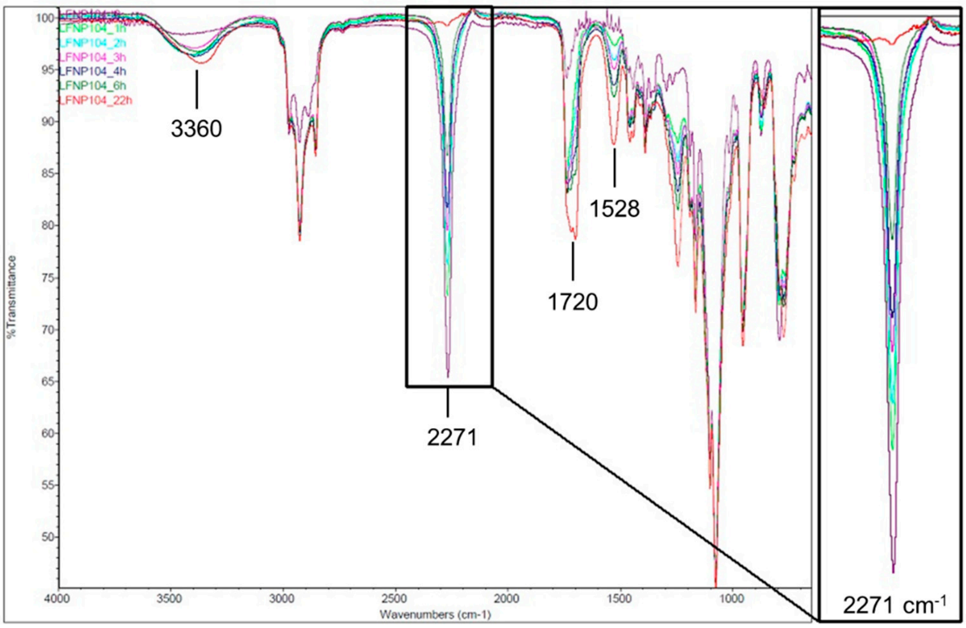

Figure S1. Infrared spectra monitoring of the glyceryl monoricinoleate silylation (IGRe (1.0) synthesis). Time = 0h (purple), 1h (light green), 2h (light blue), 3h (pink), 4h (dark blue), 6h (dark green), 22h (red)

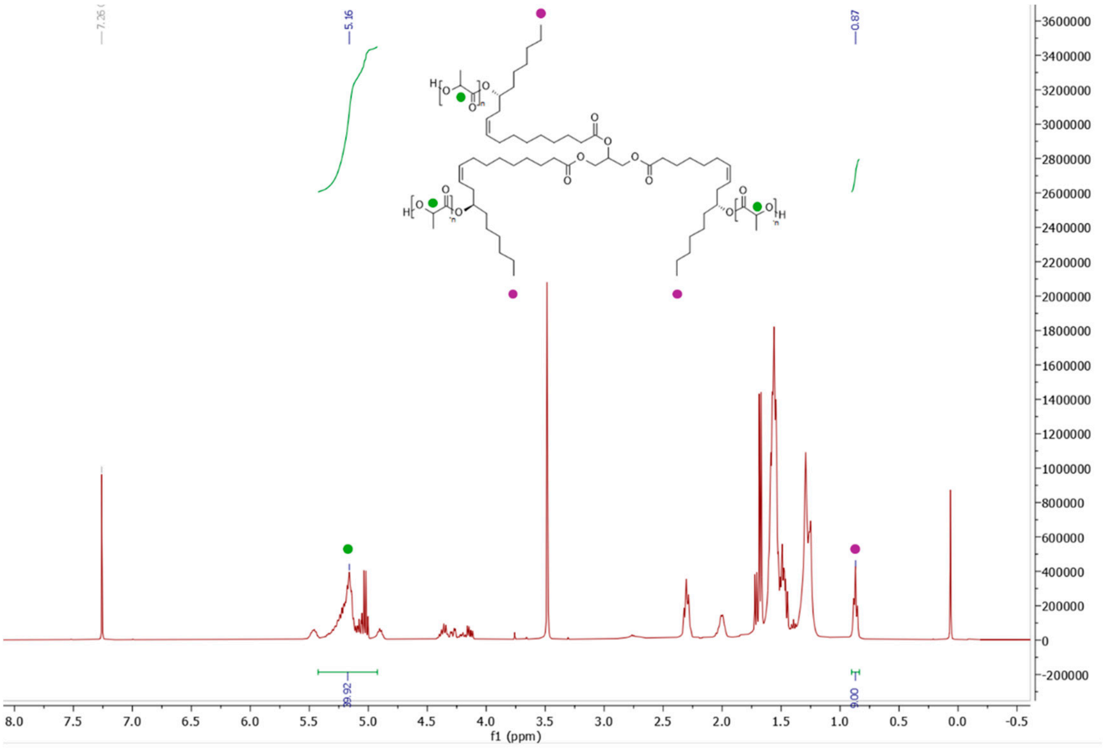

Figure S2. ^{1}H NMR spectrum of COLA. (400 MHz, $CDCl_3$). No attribution was performed as the triglyceride was not purified.

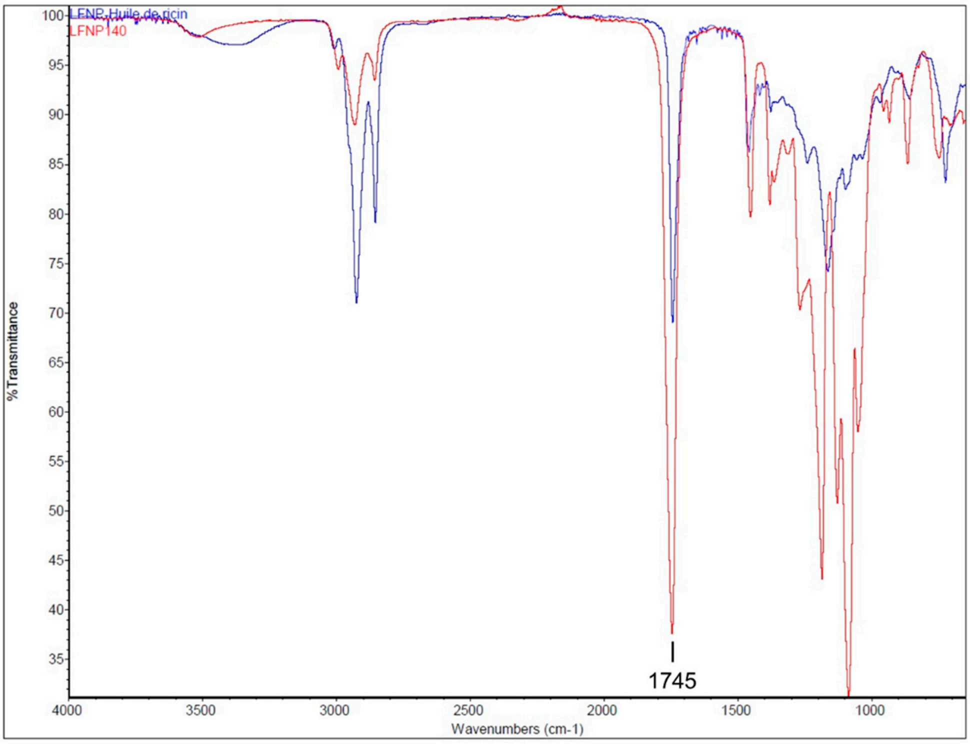

Figure S3. Infrared spectra of COLA (red) compared to castor oil (blue)

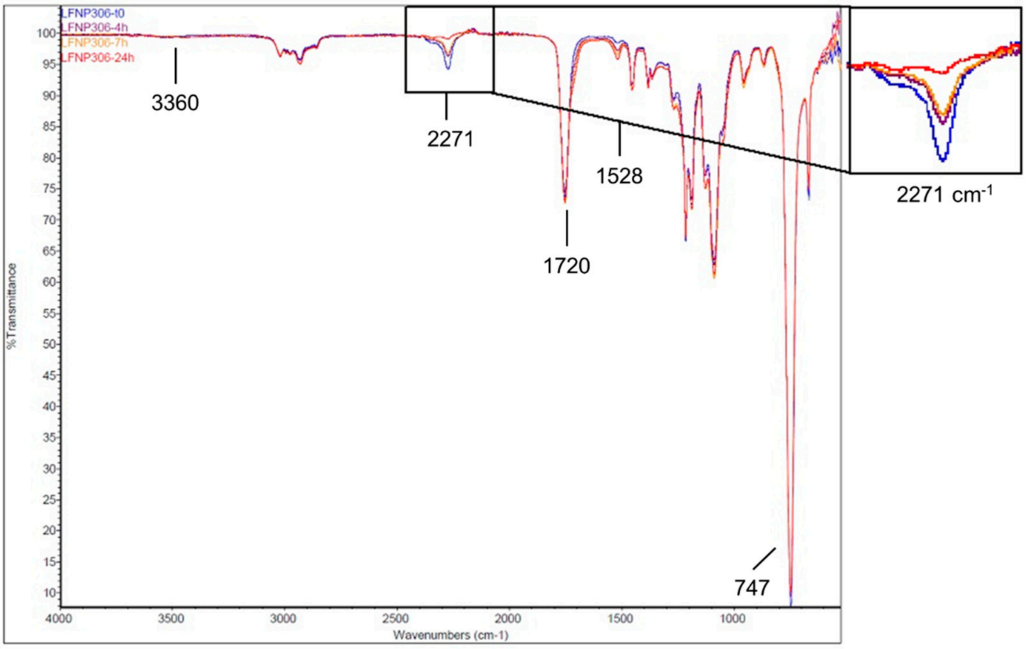

Figure S4. Infrared spectra for the monitoring of COLA silylation. Time = 0h (blue), 4h (purple), 7h (orange), 24h (red)

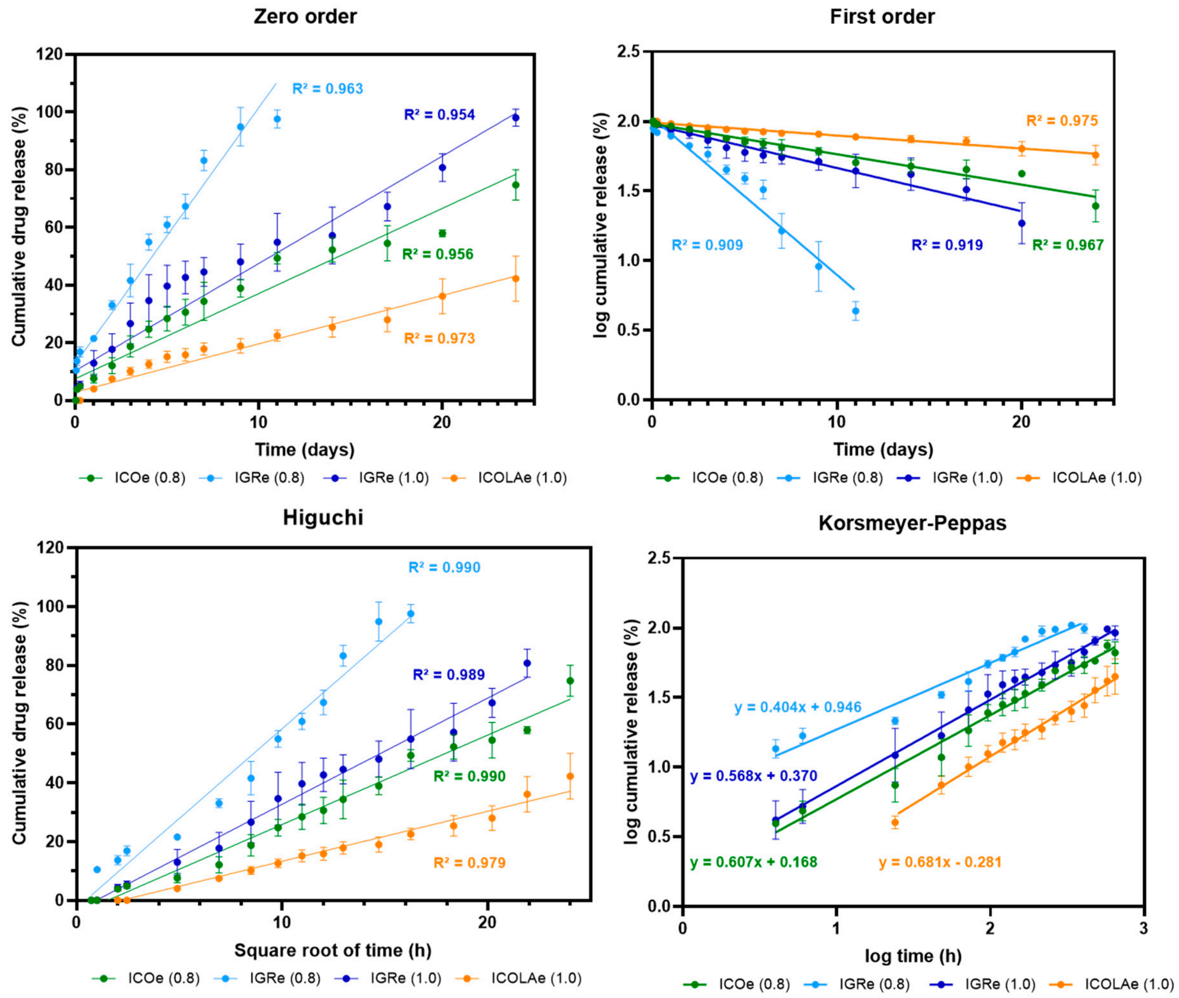

Figure S5. Release kinetic models of *in vitro* JMV5038 release tests
